# Exploring mitogenomic data to enhance the understanding of Seirinae (Collembola: Entomobryidae) evolution, distribution and taxonomy

**DOI:** 10.1186/s12983-024-00549-9

**Published:** 2024-12-04

**Authors:** Bruno Cavalcante Bellini, Nikolas Gioia Cipola, Sopark Jantarit, Nathália Michelly da Cunha Santos, Areeruk Nilsai, Hsin-Ju Cheng, Nerivânia Nunes Godeiro

**Affiliations:** 1 Department of Botany and Zoology, Biosciences Center, Federal University of Rio Grande do Norte, Natal, Rio Grande do Norte, Brazil; 2 Excellence Center for Biodiversity of Peninsular Thailand, Faculty of Science, Prince of Songkla University, Hat Yai, Songkhla, 90110 Thailand; 3 Department of Biology, Faculty of Science and Digital Innovation, Thaksin University, Phatthalung, 93210 Thailand; 4 Institute of Ecology and Evolutionary Biology, National Taiwan University, Taipei, Taiwan; 5 Natural History Research Center, Shanghai Natural History Museum, Shanghai Science and Technology Museum, Shanghai, 200041 China

**Keywords:** Alien species, Integrative taxonomy, Phylogeny, Species synonyms, Systematics

## Abstract

The subfamily Seirinae (Collembola: Entomobryidae) is among the most diverse suprageneric taxa of epedaphic springtails and is particularly abundant and species-rich in tropical and subtropical regions. Numerous studies have investigated its internal and external phylogenetic relationships, enhancing our understanding on Seirinae systematics. Recent taxonomic efforts have also advanced our knowledge on the subfamily morphology and diversity. In this study, we reevaluate the phylogeny of Seirinae using the mitogenomes of 26 samples from different continents, aiming to discuss recent findings regarding the group systematics, to identify cases of alien species introduced in China and Thailand, and propose new synonyms for *Seira* spp. Additionally, we describe a new species from Thailand and provide a redescription of *Seira brasiliana* (Arlé) based on the study of Brazilian and Chinese specimens. Through these efforts, we provide further ground for understanding the evolution, systematics, distribution and taxonomy of the group, contributing to future studies on Seirinae and Entomobryidae.

**Supplementary Information:**

The online version contains supplementary material available at 10.1186/s12983-024-00549-9.

## Background

The subfamily Seirinae *sensu* [111] (Collembola: Entomobryoidea: Entomobryidae) represents one of the most successful extant lineages of epedaphic springtails. It gathers about 230 valid species mostly distributed in tropical and subtropical regions, with a few species also known from more temperate regions, representing one of the largest subfamilies of Collembola [6, 97]. At least in the Neotropical Region, Seirinae are commonly the dominant taxa among entomobryids in warmer ecosystems [38, 39, 105]. Additionally, some species, such as *Seira dowlingi* (Wray) [98] and *S. domestica* (Nicolet) [78], are widely distributed across different continents [6, 29, 32, 48, 66, 89].

The Seirinae have recently undergone numerous revisions. A survey of the species from the Americas was presented by Christiansen & Bellinger [23]; the dorsal chaetotaxy and development patterns of *Seira* Lubbock [67] were revised in depth by Soto-Adames [90]; a new genus of Seirinae was described — *Tyrannoseira* Bellini & Zeppelini [9], while a subgenus — *Lepidocyrtinus* Börner [17] — was raised to the genus level more recently [14, 33, 46]; the subfamily systematic validity, composition and external relationships were studied by Zhang & Deharveng [108], Zhang et al. [110, 111], Godeiro et al. [49, 50] and Bellini et al. [15]; its internal relationships and genera validity were evaluated in the phylogenies of Godeiro *et al.* [46, 51, 52]; and the overall morphology of the genera was presented in detail in the revisions of Cipola *et al.* [29, 30, 32, 33]. The Seirinae were also the subject of many species descriptions in recent years, with at least 32 new species discovered and published during the last decade [3, 4, 14, 16, 27–30, 33, 44, 45, 47, 79, 80].

The use of molecular markers revolutionized the study of zoological evolution, systematics, biogeography and taxonomy. For springtails, such tools were applied, for example, to test population structuring, to support cryptic diversity, to investigate the phylogenetic validity and relationships of different taxa and to track the age of cladogenetic events [15, 25, 34, 37, 50, 87, 93, 95]. The study of population distributions, especially those observed very far from each other, has also benefited from the use of molecular tools [26]. For instance, Godeiro & Zhang [48] observed that the Chinese and Brazilian populations of *Seira dowlingi* are virtually identical from a morphological and molecular perspective, supporting that such populations not only represent the same taxon but were also likely recently introduced in China by anthropogenic actions. These observations prove the importance of integrative taxonomy.

Here, we revisit the Seirinae, focusing on the Asiatic lineages, by applying the study of molecular markers combined with morphological examination to draw further conclusions from the group. With such efforts we were able to test and discuss recent findings concerning the internal systematics of the subfamily, identify cases of Neotropical species introduced in Asia, describe a new species from Thailand, and present new synonyms of *Seira* spp., including the redescription of *Seira brasiliana* (Arlé) [1].

## Methods

### Sampling and identification

Specimens of the new species were collected by visual searching using entomological aspirators and sieving directly from debris and leaf litter. The new species was found in an orchard on Koh Phra Thong, an island located approximately 2 km off the west coast of Thailand, on the Andaman Sea. The island is separated from the mainland by an approximately 7 m deep canal situated in Khura Buri district, Phang Nga Province, southern Thailand. It has an area of ca. 102 km^2^ and is the largest island in Phang Nga Province. Koh Phra Thong is characterized by a sandbar plain area surrounded by a lagoon system with dense mangrove forest on the east coast and a long white sandy forested beach on the west coast. In the inland region, at the middle of the island, the area is covered with a swamp forest mixed with woodland-golden grassland, an ecosystem known as the Thailand savanna. The sampled specimens were kept in 95% ethanol in the field and later stored at -20°C in a freezer.

### Taxonomic description

Specimens preserved in absolute ethanol were cleared in Nesbitt’s solution and mounted on glass slides in Hoyer’s medium [59]. Specimens in ethanol gel were photographed in 70% ethanol under a Leica S8APO stereomicroscope attached to a Leica EC4 camera using LAS v. 4.12 software. Scanning electron microscope images were taken at the Scientific Equipment Center, Prince of Songkla University (Thailand). The specimens were cleaned before dehydrating in ethanol (99%) and processed by being critically dried with CO2 (CPD) in a Torusimis Autosamdri® 931 dryer. Finally, they were sputter-coated with gold using a Denton Vacuum Desk V and examined using Apreo SEM/FEI. All the photographs were subsequently improved regarding the brightness and contrast using Adobe Photoshop CS6 (Adobe Inc.).

Specimens of *S. phrathongensis* **sp. nov.** and *S. brasiliana* were deposited at the Natural History Museum, Shanghai, China (SNHM); the Collembola Collection of the Federal University of Rio Grande do Norte, Rio Grande do Norte, Brazil (CC/UFRN); the Invertebrate Collection of the National Institute of Amazonian Research, Manaus, Brazil (INPA); and the Princess Maha Chakri Sirindhorn Natural History Museum (NHM-PSU), Songkhla, Thailand, as listed in the results.

The terminology used in the descriptions is as follows: clypeal and prelabral chaetotaxy after Yoshii & Suhardjono [102], labral chaetotaxy after Cipola et al. [27], labial papillae and maxillary palp after Fjellberg [40], labial chaetae after Gisin’s system [43], postlabial chaetotaxy after Chen & Christiansen [24], as used in Cipola et al. [29], trochanteral organ after Christiansen [22] and South [91], unguiculus lamellae after Hüther [56], and manubrial ventral formula after Christiansen & Bellinger [23]. The dorsal head chaetotaxy was depicted following the system first proposed by Mari-Mutt [69], while the dorsal trunk followed Szeptycki [94], both with additions of Soto-Adames [90], Cipola et al. [29] and Zhang et al. [111]; for the specialized dorsal chaetae (S-chaetae), we followed Zhang & Deharveng [108].

**Fig. 1:**
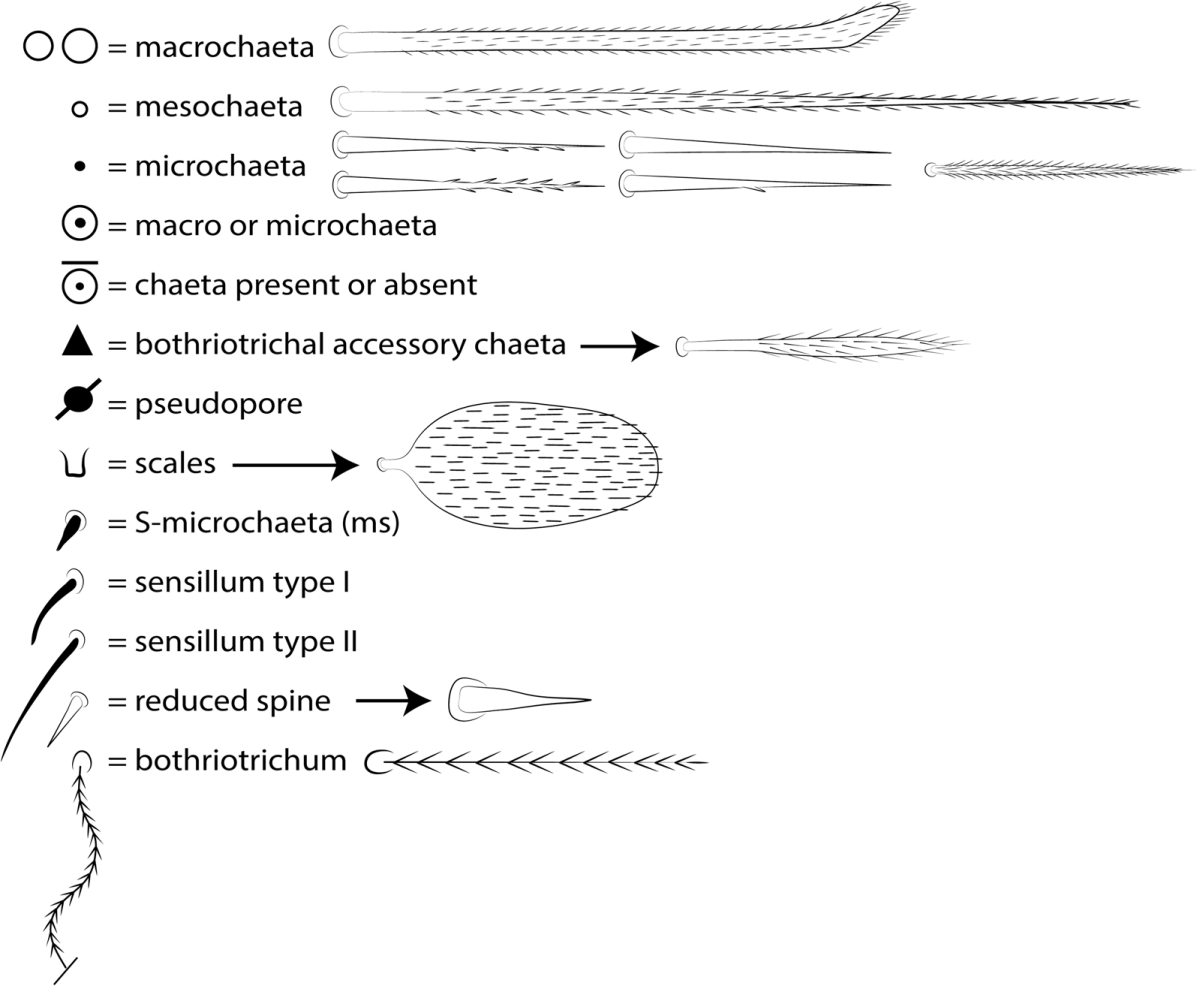
Symbols used to depict the chaetotaxy of *Seira* species.

The symbols used to depict the chaetotaxy are presented in Fig. 1. The chaetotaxy data are depicted in the text considering only one side of the body, except for the labral, prelabral and ventral manubrial chaetae. The abbreviations used in the descriptions are listed at the end of the text. Chaetal labels and other relevant structures are all marked in bold in the text.

### DNA extraction, sequencing and mitogenome assembly

One individual of *Seira phrathongensis* **sp. nov.** preserved in absolute ethanol was sent to Shanghai Yaoen Biotechnology Co., Ltd., China, where all molecular steps were performed following the kits manufacturer’s protocols. A TIANamp MicroDNA Extraction Kit (4992287 - Tiangen Co., Ltd., China) was used to extract the DNA, and a KAPA Hyper Prep Kit (07962355001 - Roche, Basel, Switzerland) was used to construct the DNA library. The Illumina NovaSeq 6000 platform produced approximately 10 Gbp of paired-end reads 150 bp long. Previous to the main analyses, a quality control of the raw sequencing data was performed to correct possible errors. These steps were performed using BBTools (sourceforge.net/projects/bbmap/), pipelines “clumpify.sh” and “bbduk.sh”. MitoZ v2.4-alpha [74] was used to assemble the raw data and annotate and visualize the mitogenome of *Seira phrathongensis* **sp. nov.** The final sequence was submitted to the NCBI nucleotide database, and the raw sequence data are publicly available at the NCBI Sequence Read Archive (SRA). The accession numbers are listed at the end of this manuscript and in Table 1.

**Table 1:** Taxonomic information of the samples and species used in the phylogenetic analyses

No	Species	Subfamily	Country	GenBank number
1	*Lepidocyrtus sotoi* Bellini & Godeiro, 2015 in [13]	Lepidocyrtinae	Brazil	MT928545.1
2	*Lepidocyrtus nigrosetosus* Folsom [41]	Lepidocyrtinae	Brazil	MW033192.1
3	*Lepidocyrtinus dapeste* (Santos & Bellini, 2018 in [14])	Seirinae	Brazil	MF716609.1
4	*Lepidocyrtinus diamantinae* (Godeiro & Bellini) [45]	Seirinae	Brazil	MF716594.1
5	*Lepidocyrtinus harena* (Godeiro & Bellini) [44]	Seirinae	Brazil	MF716617.1
6	*Lepidocyrtinus paraibensis* (Bellini & Zeppelini) [8]	Seirinae	Brazil	MF716600.1
7	*Seira atrolutea* (Arlé) [1]	Seirinae	Brazil	MF716602.1
8	*Seira atrolutea* (*S. paulae* Cipola & Bellini, 2014 in [28])	Seirinae	Brazil	MF716601.1
9	*Seira boneti* (Denis) [36]	Seirinae	China	OP181099.1
10	*Seira brasiliana* (Arlé) [1] (Paraná-PR)	Seirinae	Brazil	MF716619.1
11	*Seira brasiliana* (*S. potiguara* Bellini, Fernandes & Zeppelini [12]) (Rio Grande do Norte-RN)	Seirinae	Brazil	MF716613.1
12	***Seira brasiliana*** (***S. oligoseta Lee & Park*** [65]**) (Nanning-NAN)**	**Seirinae**	**China**	**OR804098.1**
13	***Seira brasiliana ***(***S. oligoseta*) (Xiamen-XIA)**	**Seirinae**	**China**	**OR804097.1**
14	*Seira dowlingi* (Wray) [98]	Seirinae	Brazil	MF716615.1
15	*Seira dowlingi*	Seirinae	China	MN419950.1
16	***Seira dowlingi***	**Seirinae**	**Thailand**	**NA**
17	***Seira dowlingi***	**Seirinae**	**Mexico**	**NA**
18	*Seira dollfusi* Carl [21]	Seirinae	Hungary	NA
19	*Seira ferrarii* Parona [81]	Seirinae	Hungary	OR206048.1
20	*Seira mendocae* Bellini & Zeppelini [7]	Seirinae	Brazil	MF716597.1
21	*Seira pallidipes* Reuter [84]	Seirinae	Hungary	OR115504.1
22	***Seira phrathongensis*** **sp. nov.**	**Seirinae**	**Thailand**	**PP191133.1**
23	*Seira ritae* Bellini & Zeppelini [10]	Seirinae	Brazil	MF716616.1
24	*Seira ritae* (*S. coroatensis* Godeiro & Bellini [45])	Seirinae	Brazil	MF716614.1
25	*Seira sanloemensis* Godeiro & Cipola, 2020 in [47]	Seirinae	Cambodia	MT997754.1
26	*Seira tinguira* Cipola & Bellini, 2014 in [28]	Seirinae	Brazil	MF716620.1
27	*Tyrannoseira bicolorcornuta* (Bellini, Pais & Zeppelini) [11]	Seirinae	Brazil	MF716599.1
28	*Tyrannoseira raptora* (Zeppelini & Bellini) [104]	Seirinae	Brazil	MF716610.1

Newly assembled mitogenomes are marked in bold. NA: Not available in GenBank, published in FigShare.

### Matrix generation and phylogenetic analyses

The phylogenetic tree generated in our study included 26 Seirinae samples and two outgroups (*Lepidocyrtus sotoi* Bellini & Godeiro, 2015 in [13], and *L. nigrosetosus* Folsom [41]) (Table 1). Most of the sequences were retrieved from NCBI, except for *Seira dowlingi* (Mexico), *S. dowlingi* (Thailand) and *S. brasiliana* (two samples from China), which are also newly sequenced for the present study and were assembled following the same procedures described in the previous topic. With the aim of discovering the phylogenetic position of *Seira phrathongensis* **sp. nov.** and checking the validity of two species groups from different locations (*Seira dowlingi* and *Seira brasiliana*), a phylogenetic matrix was generated following a custom pipeline. In summary, all 13 protein coding genes (PCGs) from each species were placed in different folders. TransDecoder v.5.5.0 (http://transdecoder.github.io/) was used to translate the nucleotides into amino acids. MAFFT v.7.470 software under the “L-INS-I” algorithm was used for alignment, and Trimal v.1.4.1 software [20] was used to trim the PCGs with the “-gappyout” option. FASconCAT-G v.1.04 [62] concatenated the final sequences in a matrix with 3477 amino acid sites (all codon positions) and 13 loci. Both outgroups were constrained in the beginning of the matrix prior to the analyses. The IQTree v.2.1.2 [76] was used to perform the maximum likelihood (ML) analyses, with the partitioned dataset option “MFP”, allowing ModelFinder [60] to choose the best substitution model for each partition. One thousand UFBoot2 [55] replicates were run for the ML analyses. Details about the models and partitions are presented in the Additional File 1. Bayesian inference analyses (BI) were performed with PhyloBayes-MPI v.1.8 [64] using the CAT + GTR site-heterogeneous mixture model. The dataset used was the same utilized for ML analyses containing amino acids sequences of the 13 loci. Two independent Markov chain Monte Carlo (MCMC) chains were run and terminated when the runs converged (maxdiff < 0.1). No tree constraints were used. Ten percent of the generated trees were removed as burn-in, and a consensus tree was calculated from the remaining trees, using the “bpcomp” command. The resulting trees for the ML and BI inferences were visualized in FigTree v.1.3 (http://tree.bio.ed.ac.uk/software/figtree/).

### Species delimitation analyses

To determine the putative species boundaries between all analyzed taxa from our study, we used the Poisson tree processes (PTP) model on a non-ultrametric tree. PTP analysis delimits species based on the phylogenetic species concept and detects significant differences in the number of substitutions between different species and within the same species, resulting in a phylogenetic tree with bootstrap values, delimitating operational taxonomic units (OTUs) [107]. To perform the analysis, we exported the resultant tree from our ML analysis to the Newick format and used it as input in the online platform (https://species.h-its.org/ptp), with the default parameters (100,000 generations; Thinning: 100; Burn-in: 0.1). To confirm the PTP delimitation results, we also prepared a distance matrix in MEGA v.X [63, 92] using the final amino acid alignment of the 13 PCGs. The variance was estimated using 1000 bootstrap replications, uniform rates, and fewer than 95% alignment gaps, missing data, and ambiguous bases were allowed at any position (partial deletion option).

## Results

### Mitogenome of the new species

MitoZ recovered a linear sequence 14,598 bp in length corresponding to the mitogenome of *Seira phrathongensis* **sp. nov. **The region between ND3 and ND5 was not well assembled, causing the absence of four tRNA genes (Ala, Arg, Glu, and Ser1). Other genes are arranged in the gene order typical of Pancrustacea, and the same arrangement was found in the majority of the Collembola representatives. Canonical initiation codons (ATA or ATG), encoding the amino acid methionine, are present in six protein-coding genes (PCGs), namely, ND2, COX2, ATP6, COX3, ND3, and CYTB, whereas the other seven genes start with nonstandard codons (ATT or TTG) (Table 2). All PCGs had complete stop codons (TAA or TAG) (Table 2).

**Table 2:** Order and features of mitochondrial protein-coding genes and ribosomal RNAs of *Seira phrathongensis* **sp. nov.**

Start	End	Length (bp)	Direction	Start/End code	Gene name
305	1304	1000	+	ATG/TAA	ND2
1498	3031	1534	+	ATT/TAA	COX1
3097	3781	685	+	ATA/TAA	COX2
3916	4075	160	+	ATT/TAG	ATP8
4068	4749	682	+	ATG/TAA	ATP6
4751	5539	789	+	ATG/TAA	COX3
5614	5962	349	+	ATA/TAA	ND3
6228	7920	1693	-	TTG/TAG	ND5
8004	9330	1327	-	TTG/TAG	ND4
9350	9632	283	-	ATT/TAA	ND4L
9767	10250	484	+	ATT/TAA	ND6
10249	11386	1138	+	ATG/TAA	CYTB
11523	12435	913	-	ATT/TAG	ND1
12473	13746	1274	-		l-rRNA (16S)
13766	14511	746	-		s-rRNA (12S)

### Seirinae phylogeny and species delimitation analysis

**Fig. 2:**
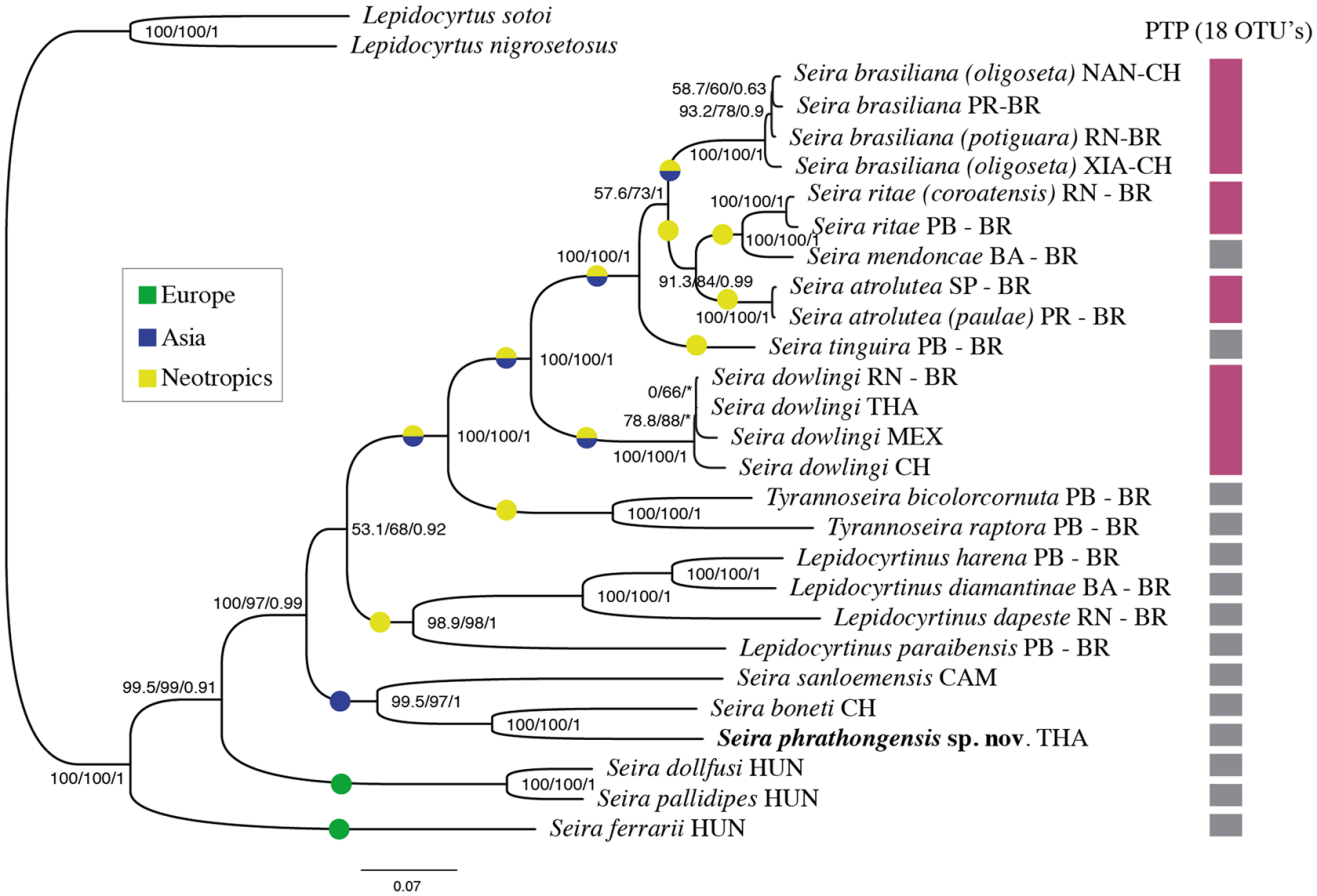
Phylogeny of world Seirinae, indicating the placement of *Seira phrathongensis* **sp. nov.** (in bold). Tree constructed based on maximum likelihood (ML) and Bayesian inference (BI) analyses of mitochondrial genomes. The numbers at the nodes represent the SH-aLRT support and bootstrap values (both for ML) and the posterior probability (BI support), respectively; ‘*’ represents a polytomy in BI; lateral bar represents the Operational Taxonomic Units (OUT’s) defined by PTP species delimitation analysis (Additional Files 2 and 3). Colored circles mark the origins of each studied sample.

The relationships of the sampled taxa are summarized in Fig 2. The overall BI node support was very high (0.99) or absolute (1) for most branches, except for the internal groupings of *S. brasiliana* and *S. dowlingi* sampled populations; the basal branches clustering the Neotropical Seirinae; and *S. ferrarii* Parona [81] with all other Seirinae. The ML overall support values (SH-aLRT and bootstrap) were also mostly high (≥95) or absolute (100), with some exceptions, but the ML support was mostly lower than that of the BI analysis.

Our tree recovered *S. ferrarii* as the most basal sampled lineage of Seirinae, followed by the grouping of *S. pallidipes* Reuter [84] + *S. dollfusi* Carl [21], all three from Europe. The Asiatic species were subsequently grouped together following the topology: *S. sanloemensis* Godeiro & Cipola, 2020 + (*S. phrathongensis* **sp. nov.** + *S. boneti* (Denis [36])), and as the sister group of the Neotropical Seirinae, recovered as *Lepidocyrtinus* + (*Tyrannoseira* + Neotropical *Seira*). Specimens of *Seira oligoseta* Lee & Park [65] from China, and *S. dowlingi* from Thailand and China were mixed with Neotropical populations of *S. brasiliana* and *S. dowlingi*, respectively (Fig. 2).

Concerning the PTP species delimitation analysis, all sampled populations of *S. dowlingi* were recovered as belonging to the same species, similar to *S. oligoseta*, *S. potiguara* Bellini, Fernandes & Zeppelini [12] and *S. brasiliana* populations. The Brazilian *Seira coroatensis* Godeiro & Bellini [45] also clustered with *S. ritae* Bellini & Zeppelini [9, 10], while *S. paulae* Cipola & Bellini, 2014 in [28] grouped with *S. atrolutea* (Arlé) [1] (Fig. 2). The bPTP results are based on BI, and they imply that our dataset has 18 OTUs (excluding the outgroups), with the posterior probability support for each species group detailed in the Additional File 2. The distance matrix generated by MEGA to detect the number of amino acid differences per sequence from between sequences corroborated the bPTP delimitation analysis, with the species delimited as same having at most 90 different amino acids in the 13 PCGs alignments. This number was a minimum of 220 when comparing different species (see the Additional File 3).

**New species description**

 **Class Collembola** **Lubbock** [67]

**Order** **Entomobryomorpha** **Börner** [18]

 **Entomobryidae** **Tömösváry** [96]

**Subfamily Seirinae Yosii **[100] ***sensu*** [111]

**Genus *Seira*** **Lubbock** [67]

*Seira phrathongensis* **sp. nov.** Bellini, Godeiro, Cipola & Santos

**Fig. 3:**
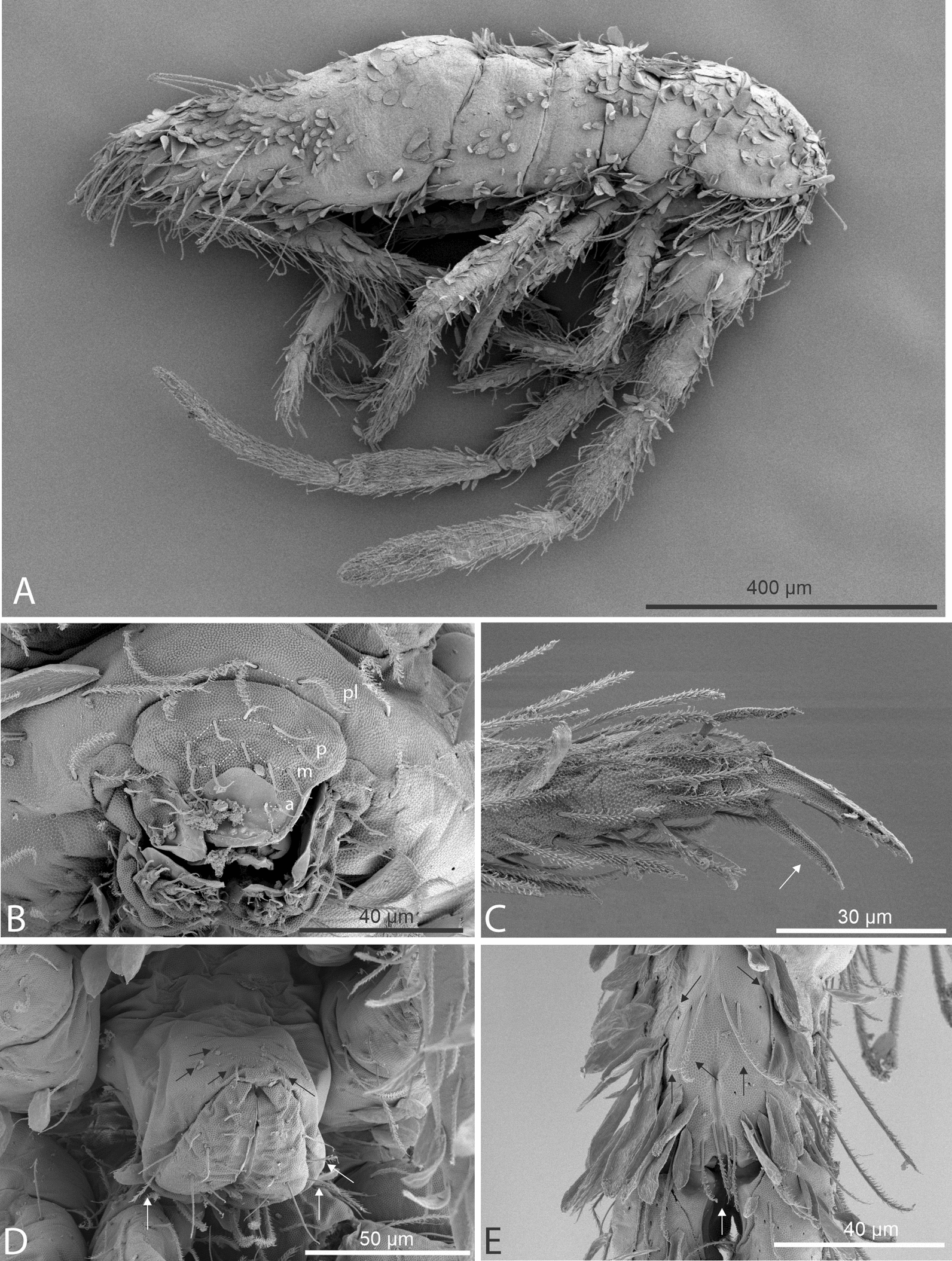
SEM images of *Seira phrathongensis* **sp. nov.**: **A**, Habitus (lateral view); **B**, External mouthparts showing the prelabral and labral chaetae (frontal view); **C**, Empodial complex III (posterior view, tenent-hair is missing), the white arrow points to the smooth postero-external lamella of unguiculus; **D**, Ventral tube (ventro-posterior view), black arrows point to the smooth posterior chaetae of the corpus, while the white arrows point to ciliate chaetae on the lateral flaps; **E**, Distal manubrium (ventral view), the white arrow points to the distal internal shorter chaetae, and the black arrows mark the subapical chaetae, including the shorter lateral ones.

**Fig. 4:**
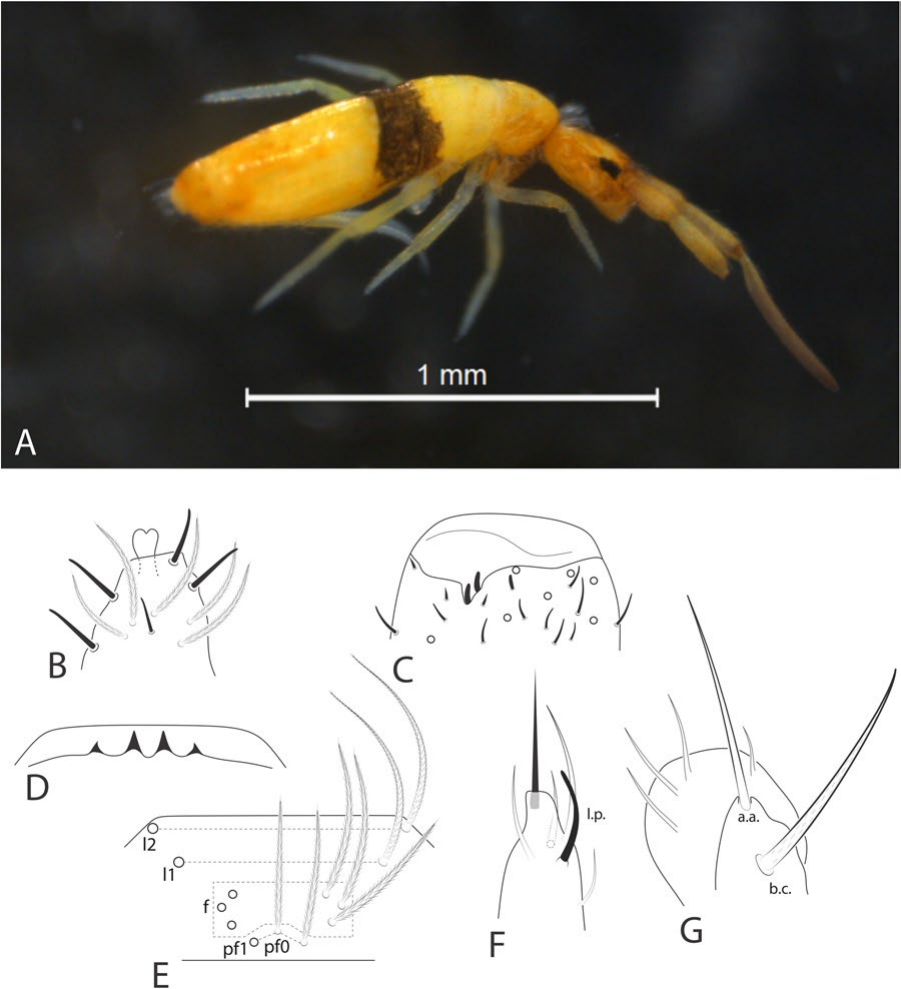
*Seira phrathongensis* **sp. nov.** habitus and head structures: **A**, Habitus of a fixed specimen in ethanol (lateral view); **B**, Distal left Ant. IV (dorsal view); **C**, Distal left Ant. III (ventral view); **D**, Labral papillae (dorsal view); **E**, Clypeal chaetotaxy (dorsal view); **F**, Right labial papilla E (ventral view); **G**, Right maxillary outer lobe (ventral view).

**Fig. 5:**
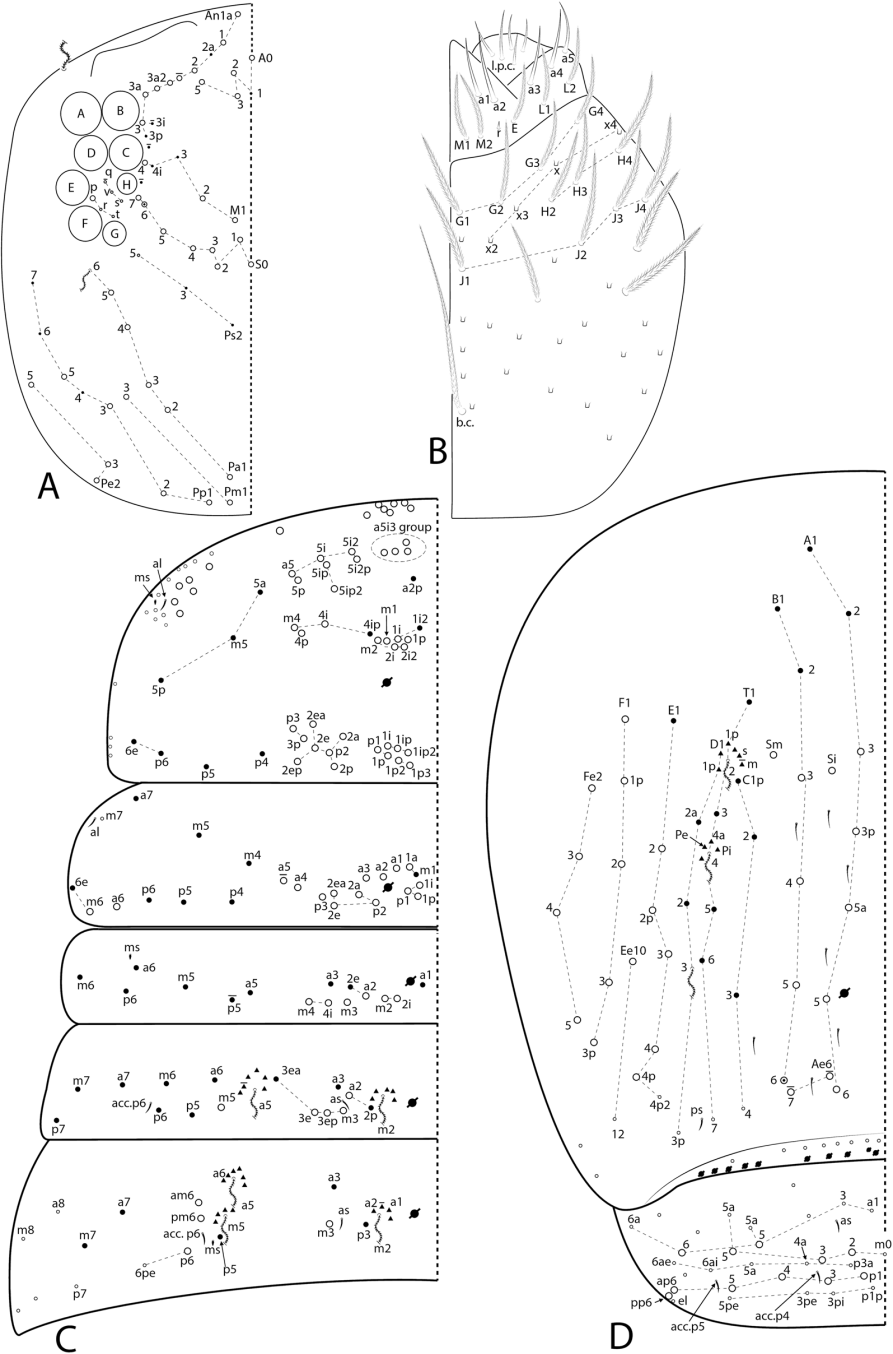
*Seira phrathongensis* **sp. nov.** main chaetotaxy: **A**, Dorsal head and eyes (left side); **B**, Ventral head labial and postlabial regions (right side); **C**, Dorsal Th. II–Abd. III (left side); **D**, Dorsal Abd. IV–V (left side).

**Fig. 6:**
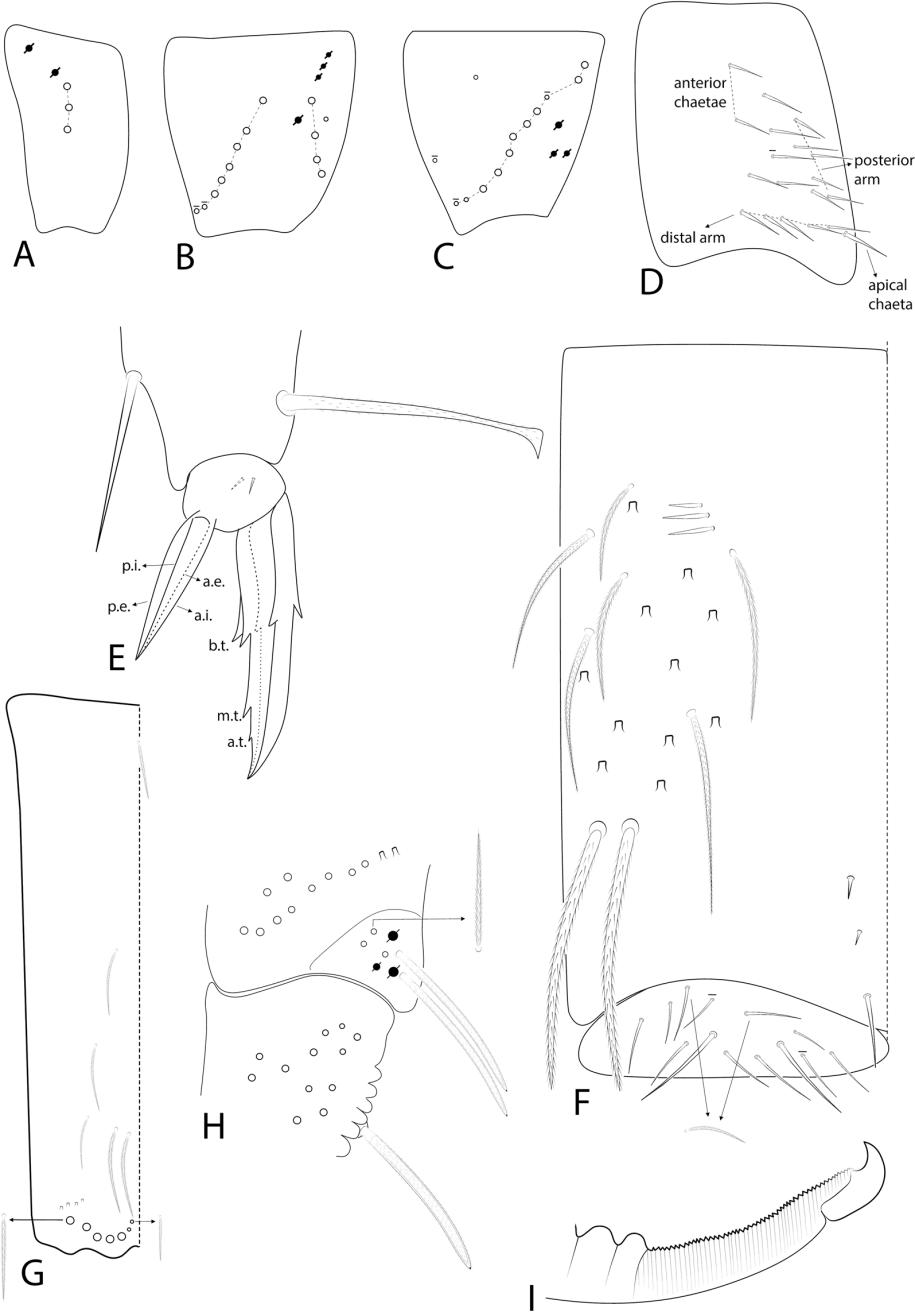
*Seira phrathongensis* **sp. nov.** trunk appendages: **A–C**, Coxae I–III, respectively (outer view); **D**, Trochanteral organ (posterior view); **E**, Empodial complex III (posterior view); **F**, Ventral tube (lateral view, anterior face to the left), arrows point to chaetae which can also be ciliate; **G**, Ventral manubrium (left side); **H**, Left manubrium and dens (dorsolateral view); **I**, Distal dens and mucro (outer view).

Figs 3, 4, 5, 6, Table 3

**Table 3.** Seirinae species recorded from Asia with six mac on Abd. I

Species/features	L. schaefferi*^,2,4^	*S. atlantica*^5^	*S.. boneti*^9^	*S. domestica*^8^	*S. hazrai*^6^	*S. prabhooi*^7^	*S. simbalwaraii*^7^	*S. thailandica*^1^	*S.tigridica*^3^	*S. phrathongensis ***sp. nov.**
Color pattern (dark pigments distribution)	At least on proximal legs, distal Abd. II, most Abd. III, and a lateral spot on distal Abd. IV	At least on Ant. I–III, frontal and posterior, head, proximal legs and lateral Abd. IV	Usually with spots on Th. III–Abd. I and lateral Abd. IV	On distal half of Ant II to apex of Ant IV	At least on anterior Th. II, few spots on Th. III and Abd. I and scattered on Abd. II–III	Distal Ant. III–IV and dorso-frontal head	Ant. IV, apical Ant. II–III, mouth, Abd. I–III, posterior Abd. IV and ventral abdomen	Antennae distally, frontal head, proximal legs; lateral Th. III, distal Abd. IV and middle Abd. V	pale	Distal Ant. II–III, proximal leg I, Abd. II anteriorly and whole Abd. III
Dorsal head **An** mac	8	10	8	11	8–9	6–7?	11–12	?	9	7–8
Dorsal head **S4** mac	+	-	+	-	-	-	-	?	-	+
Dorsal head **S5** mac	+	+	+	+	+	-	-	?	+	+
Dorsal head **S6i** mac	-	+	+	+	+	-	-	?	+	+/-
Dorsal head **Pa2** mac	-	+	+	+	+	+	+	?	-	+
Dorsal head **Pa4** mac	-	-	+	-	+	+	+	?	-	+
Dorsal head posterior mac (other than **Pa5**)	+	+	+	+	+	+	+	?	?	+
Dorsal head **Ps2** mac	-	+	-	+	+	-	-	?	+	-
Labral external papillae	developed	developed	developed	developed	developed	vestigial?	?	?	developed	reduced
Labial **r** chaeta	reduced (s)	regular (c)	reduced (s)	regular (c)	regular (s)	reduced (s)	reduced (s)	?	regular (c)	reduced (s)
Mesothoracic hood	-	-	+	-	-	-	-	-	-?	-
Th. II **a5i3** complex mac	3	-	4	-	-	-	3	?	-	4
Th. II **m1–2** complex mac	6	4	6	4	4	5	6	?	4	6
Th. II **m4** complex mac	2	3+1(**a5ip**)	3	2–3+1(**a5ip**)	3	3	3	?	3+1(**a5ip**)	3
Th. II **p2e–p3** complex mac (**PmC** region)	3^4^,5^2^	5	6–7	8–10	5	7	9	4	5	5
Th. II **p5** mac	+	+	-	+	+	-?	-	-?	+	-
Th. III central mac	14^4^,15^2^	15	14	11–15	14	13	12	14	14	13–14
Abd. II central mac	4	5	4	5	4	4	4	4	4	4
Sexually dimorphic first pair of legs	-?	-?	-	+	-?	-?	-?	-?	-?	-
Trochanteral organ spines	?	>30	16–18	20	7	11	25	12	?	18–19
Unguiculus p.e. serrations	-	+**	+	+	+	?	+	?	-	-
Ventral tube anterior chaetae	?	~18	7–10ch+3spn	10	?	2	5	2ch+3spn	?	8ch+3spn
Ventral tube lateral flap chaetae	8s+7c	17c	4s+9c	3s+16c	?	4	8–10	At least 4ch	?	9**–**13s+0–2c
Ventral tube posterior chaetae	1s	?	1c+3spn	8c	?	-	0–2(s?)	1s+6spn	?	1s+2spn
Manubrial ventro-external subapical chaetae compared to the internal ones	anterior	-	posterior	posterior	?	?	anterior	?	?	aligned or posterior
Manubrial plate	At least 2ch	?	4ch+2psp	6–7ch+3psp	4ch+2psp	?	5ch+2psp	-	?	5ch+3psp
Manubrium dorsal blunt mac	2	-?	-	-	-?	-?	-	-	-?	2
Dens dorsal blunt mac	1	-?	-	-	-?	-	-	-	-?	1
Distribution	New Guinea, Java, Philippines, Indonesia	Iran	Vietnam, Cambodia, Chna	Euro-Mediterranean region, Iran, Australia	India	India	India	Thailand	Iraq	Thailand

Morphology based on: ^1^Yosii [101],^2^Gapud [42],^3^Rusek [86],^4^Yoshii & Suhardjono [103],^5^Negri *et al.* [77]; ^6^Baquero *et al.* [3], ^7^Baquero *et al.*[4],^8^Cipola *et al.* [29],^9^Godeiro *et al.* [51]. Species distribution based on previously cited papers plus Greenslade [54] and Mayvan et al. [73]. Legends: (+) present,(-) absent; (/) or; (~) approximately; (>) more than; (?) unknown/unclear; (c) ciliate; (s) smooth; (ch) chaetae; (psp) pseudopores; (spn) spines; *Lepidocyrtinus schaefferi was depicted by Yoshii & Suhardjono [102] with five mac on Abd. I, but with six by Gapud [42],**with a single proximal serration as an external tooth.

Type material. HOLOTYPE: female on slide (CC/UFRN): Thailand: Phang Nga Province, Khura Buri District, Koh Pra Thong Island (09°02'02.3"N 98°16'13.6"E), 10 asl., from litter and debris in orchard plantation, entomological aspirator, A. Nilsai leg, 8 November 2017. PARATYPES: same data as holotype, one male on slide (CC/UFRN); one female and two males on slides (SNHM-th1.01 to SNHM-th1.03) deposited at the SNHM; and one female and one male on slides (INPA-CLL 000381–82) deposited at INPA.

Additional material. same data as holotype, six specimens (in ethanol) deposited at the NHM-PSU.

Diagnosis. Abd. II posteriorly and whole Abd. III dark purple, forming a transverse band. Dorsal head **An** series with 7–8 mac, **S4**–**5** and posterior head mac (other than **Pa5**) present, **Pa2** and **Pa4** as mac. Labial **r** chaeta smooth and reduced. Mesothoracic hood underdeveloped, Th. II **a** (excluding the anterior collar), **m** and **p** series with 11, 9, 15 mac, respectively, **p5** mac absent. Th. III–Abd. III central mac as 13–14, 6, 4, 1, respectively. Legs without any clear sexual dimorphism, tibiotarsus III without remarkably long chaetae, trochanteral organ with 18–19 spines, ungues lateral teeth regular, not enlarged, unguiculi **p.e.** lamella smooth. Ventral tube anteriorly with eight ciliate chaetae and three proximal spines, posteriorly with one smooth regular chaeta and two spines, lateral flap with 9**–**13 smooth and 0**–**2 ciliate chaetae. Manubrium ventral chaetae formula from the basis to the subapical region as 1, 2, 2, 2/4, ventro-external subapical chaetae posterior or aligned to the internal ones, manubrial plate with two, and dens proximally with one blunt mac, respectively.

Description. Holotype (female) with 2.26 mm, one paratype (male) with 1.27 mm. Habitus typical of *Seira* (Figs 3A, 4A). Specimens ground color pale or yellowish, with distal Ant. II–III and coxa I weakly purple (coxa I also pale in some specimens), dorso-frontal head with a typical dark spot, Abd. II anteriorly and whole Abd. III dark purple, forming a transversal band; eyepatches black (Fig 4A). Scales present on Ant. I to proximal Ant. III, dorsal and ventral head, dorsal trunk, legs, anterior ventral tube, dorsal and ventral manubrium and ventral dens (Figs 3A, 3E).

Head. Ratio of Ant. I–IV in one paratype (holotype without complete antennae): 1:1.28:1.5:2.22. Ant. IV apical bulb distally bilobed (Fig. 4B), Ant. III apical organ with two sensory rods, three short guard sens, one subapical thick and blunt sens and at least 10 surrounding sens (Fig. 4C). Labral papillae present, internal ones conical and developed, lateral ones reduced (Fig. 4D). Labral **p**/**m**/**a** rows with 5/5/4 smooth chaetae respectively, **p** row chaetae slightly longer than the others, **a** row chaetae as thick as or slightly thinner than **m** chaetae, pre-labral chaetae ciliated (Fig. 3B). Clypeal chaetotaxy as in Fig. 4E, all chaetae ciliate, **pf** chaetae slightly shorter than the **f** ones, **l1**–**2** long, strongly acuminate and slightly ciliated. Mandibles and maxillae without any clear modifications, labial papillae formula of the guards as **H**+2, **A**+0, **B**+5, **C**+0, **D**+4, **E**+4 plus a finger shaped **l.p.** surpassing the apex of the papilla (Fig. 4F). Maxillary outer lobe **b.c.** subequal to **a.a.**, sublobal plate with four chaeta-like appendices (Fig. 4G). Dorsal head with 7–8 **An**, 4 **A**, 3 **M**, 7–8 **S**, 5 **Pa**, 2 **Pm**, 4 **Pp** and 3 **Pe** mac; 8+8 eyes with 5–6 interocular chaetae, chaetal homologies and further details depicted in Fig. 5A. Labial proximal region with five smooth chaetae, labial basal chaetae formula as **M1**–**2rEL1**–**2**/**a1**–**5**; post labial region with ciliate chaetae and scales, anterior region with chaetal formula **G1**–**4**/**H2**–**4**/**J1**–**4**, **x** series with four scales, basal chaeta very long and slightly ciliate, further details depicted in Fig. 5B.

Trunk chaetotaxy. Mesothoracic hood underdeveloped (Figs 3A, 4A). Formula of the internal mac from Th. II (excluding the collar) to Abd. IV as 35, 13–14|6,4,1,10–13; of the external mac as: 0,2|0,1,3,15; and of the bothriotricha as 0,0|0,2,3,3. Abd. IV with eight posterior mes next to about 10 psp; Abd. V with 11 mac; formulae of the tergal sens from Th. II to Abd. V as 1,1|0,2,2,+,3; and of **ms** as 0,0|1,0,1,0,0. All chaetal homologies and further details depicted in Figs 5C–D. Ratio Abd. III–IV in the midline of the holotype as 1:4.6.

Trunk appendages. Leg I without any clear sexual dimorphism. Coxae I–III with 3, 11–13, 11–13 ciliate chaetae and 2, 4, 3 psp, respectively, structures positioning as in Figs 6A–C. Trochanteral organ with 18–19 smooth spine-like chaetae disposed as in Fig. 6D. Tibiotarsi without remarkably long chaetae as seen in Neotropical *Lepidocyrtinus* taxa (Fig. 3A), tenent-hairs capitate and slightly ciliate; ungues with seven teeth, one dorsal, two paired lateral and four internal, lateral teeth not enlarged, **a.t.** reduced, **m.t.** subequal to **b.t.**; unguiculi lanceolate, with all lamellae smooth (**a.e.**, **a.i.**, **p.i.** and **p.e.**) empodial complex III ratio of unguiculus, ungues, smooth chaeta and tenent-hair of the holotype as 1:1.6:1.1:1.5 (Figs 3C, 6E). Ventral tube anteriorly scaled, with two distal mac, six ciliate proximal chaetae and three smooth spines; posteriorly with two reduced smooth spines and one smooth chaeta; lateral flap with about 11–13 chaetae, 9**–**13 smooth and 0–2 ciliate (Figs 3D, 6F). Tenaculum rami with four teeth, corpus with a single ciliate chaeta. Manubrium ventral chaetae formula from the basis to the apex as 1, 2, 2, 2/4 (subapical) and 14 (apical) chaetae, ventro-external subapical chaetae posterior or aligned to the internal ones (Figs 3E, 6G). Manubrial plate with five chaetae, two of them as blunt mac, plus three psp; dens dorso-proximal region with an internal blunt mac, larger than the ones in the manubrial plate and on a small papilla (Fig. 6H); mucro typically falcate (Fig. 6I). Ratio manubrium: mucrodens of the holotype as 1:1.57.

Etymology. The new species was named after its type locality, Koh Phra Thong (Koh = Island), in Phang Nga Province, Thailand.

Habitat. *Seira phrathongensis* **sp. nov.** was collected from an orchard near the southernmost part of the island. They were found in the debris and litter near a farm pond. The region belongs to Good’s biogeographic zone 18 from the Paleotropical Region [53]. Its climate is characterized by an equatorial monsoon (Am) climate characterized by marked wet and dry seasons [61].

Remarks on the species. This is the third nominal species of *Seira* recorded in Thailand. The first was *S. thailandica* Yosii [101] found in the soil habitat of Chiang Mai, northern Thailand [101]. Recently, *S. dowlingi* was discovered in lowland forest litters (ca. 60 meters asl.) at Songkhla Province. In fact, *Seira* spp. were also recorded in Thailand from cave environments where bat guano is present and in high mountains at 700 m asl. of Doi Inthanon forest, Chiang Mai [58]. For the taxonomical remarks, see the discussion topic.

### New synonyms for *Seira* spp.

After our results (Fig. 2, Additional File 2), we herein synonymize *S. oligoseta* and *S. potiguara* with *S. brasiliana*, presenting below a redescription of the species based on our studied specimens from Brazil and China. We also synonymize *S. coroatensis* with *S. ritae*; and *S. paulae* with *S. atrolutea* (further notes ahead).

*Seira* *brasiliana* **(Arlé)** [1]

Figs. 7, 8, 9.

**Fig. 7:**
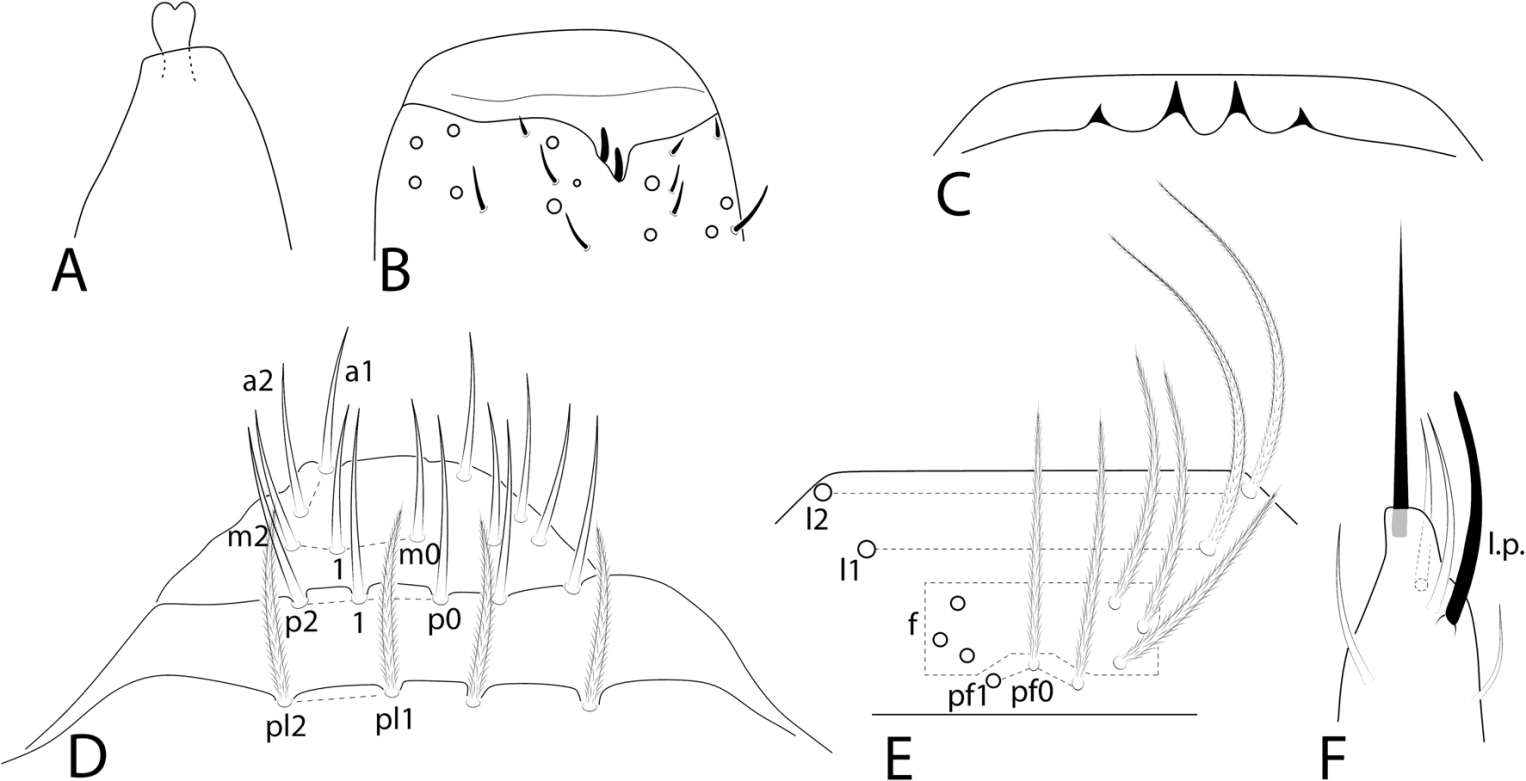
*Seira brasiliana* head structures: **A**, Distal right Ant. IV (dorsal view – chaetae omitted); **B**, Distal right Ant. III (ventral view); **C**, Labral papillae (dorsal view); **D**, Labral and prelabral chaetotaxy; **E**, Clypeal chaetotaxy (dorsal view); **F**, Right labial papilla E (ventral view).

**Fig. 8:**
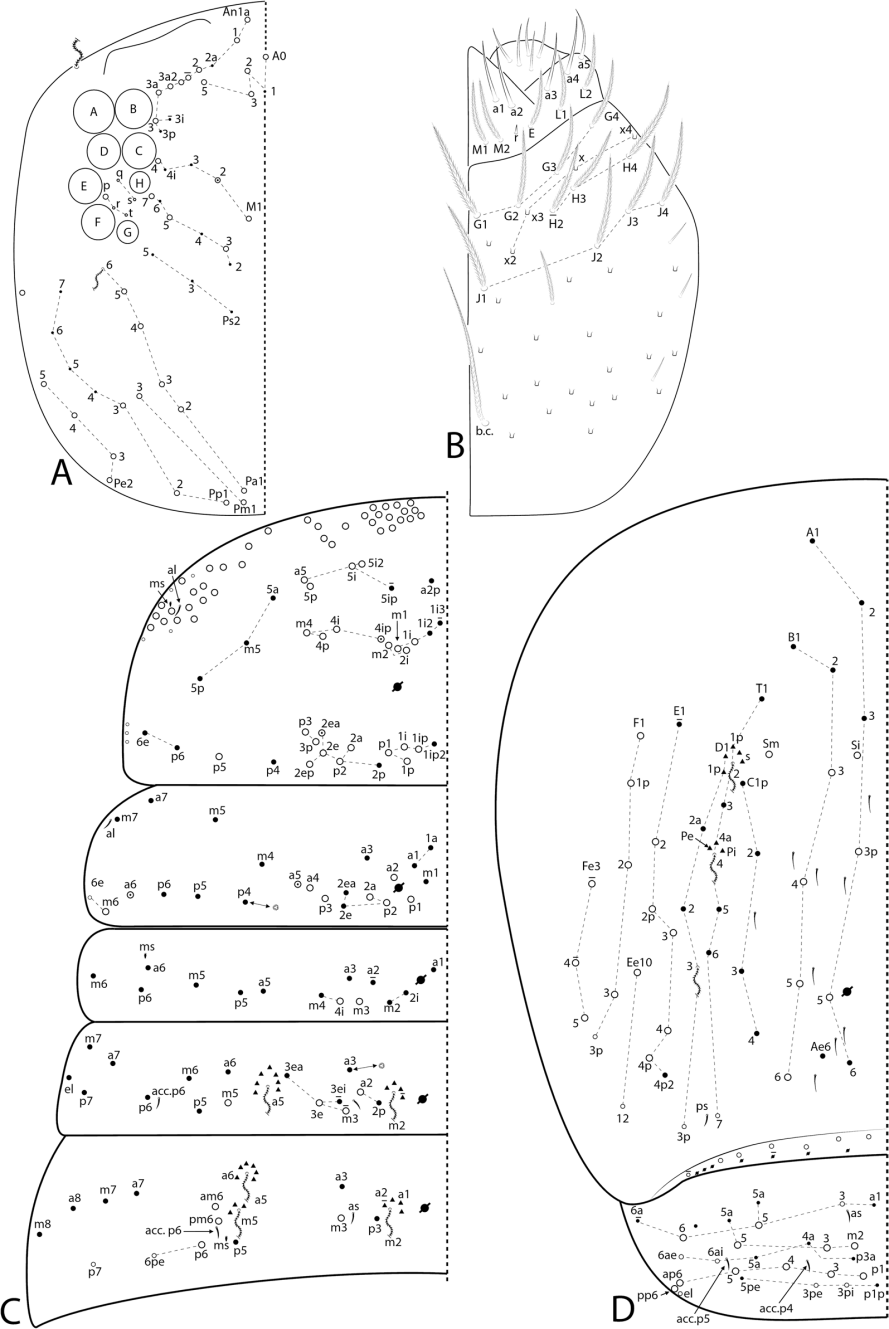
*Seira brasiliana* main chaetotaxy: **A**, Dorsal head and eyes (left side); **B**, Ventral head labial and post labial regions (right side); **C**, Dorsal Th. II–Abd. III (left side); **D**, Dorsal Abd. IV–V (left side).

**Fig. 9:**
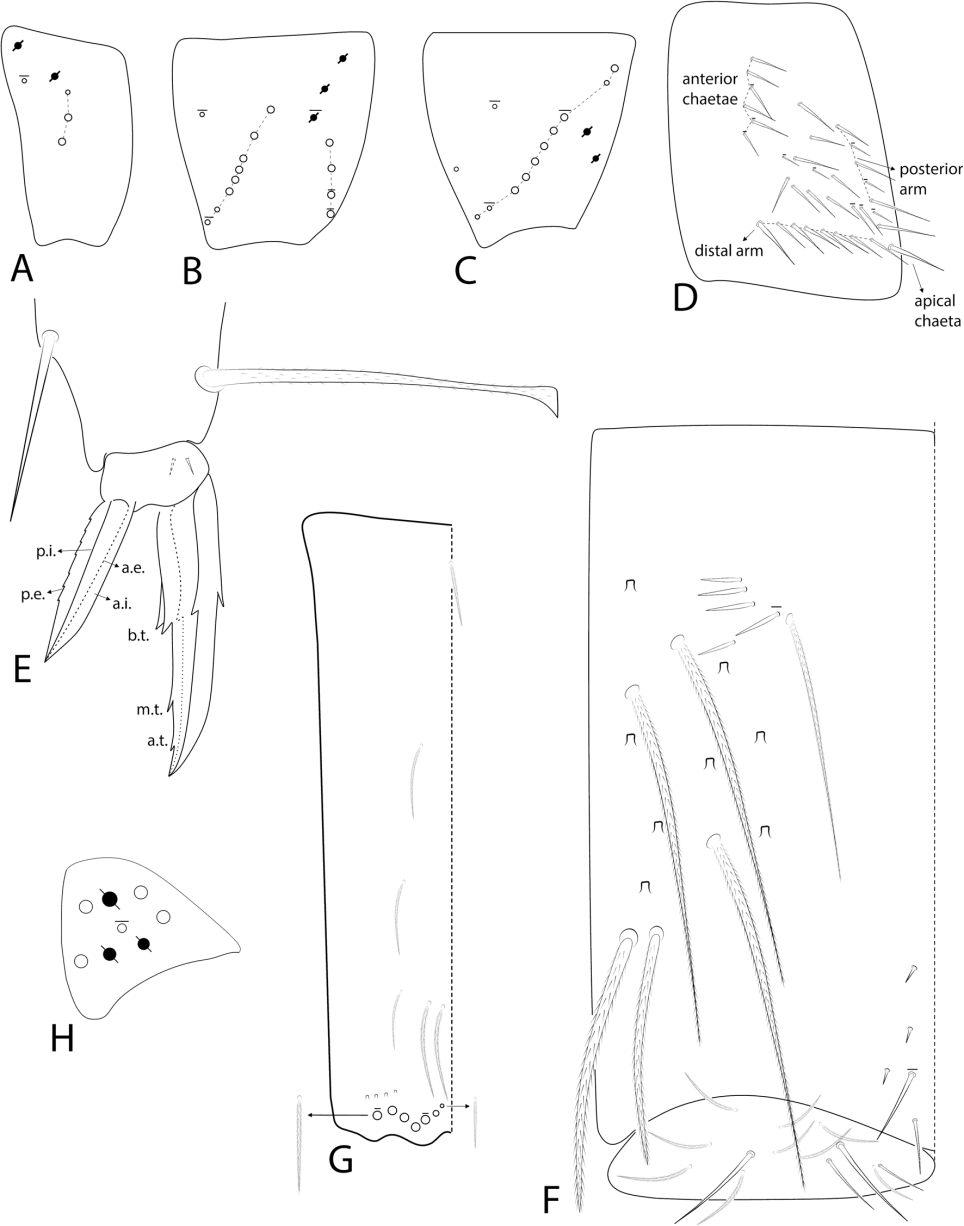
*Seira brasiliana* trunk appendages: **A–C**, Coxae I–III, respectively (outer view); **D**, Trochanteral organ (posterior view); **E**, Empodial complex III (posterior view); **F**, Ventral tube (lateral view, anterior face to the left); **G**, Ventral manubrium (left side); **H**, Right manubrial plate (dorsal view).

*Pseudosira brasiliana* Arlé [1]: 297–299, figs 21–28, Brazil, São Paulo and Mato Grosso states.

*Pseudosira brasiliana var. marginata* Arlé [1]

*Lepidocyrtinus subannulatus* Denis [35] *sensu* [70]

*Seira oligoseta* Lee & Park [65] **syn. nov.**

*Seira potiguara* Bellini, Fernandes & Zeppelini [12] **syn. nov.**

Analyzed material. Seven specimens in slides and 15 specimens in alcohol (SNHN): China: Fujian Province, Xiamen city, Xiamen Park (24°29'51.8"N 118°05'52.4"E), entomological aspirator, Qi Bao leg, 22 May 2022. One specimen (male) in slide (CC/UFRN): China: Fujian Province, Xiamen city, Xiamen Park (24°29'51.8"N 118°05'52.4"E), entomological aspirator, Qi Bao leg, 22 May 2022. Four specimens in slides and 10 specimens in alcohol (SNHN): China, Guangxi Province, Weizhou island (21°01'32.9"N 109°05'24.7"E), entomological aspirator, Godeiro, N.N., Bu, Y., Jin, Y., Qi, Y. leg, 24 September 2022. One specimen in slides and five specimens in alcohol (SNHN): China, Guangxi Province, Beihai city, Guanling Park (21°27'04.3"N 109°03'25.9"E), entomological aspirator, Godeiro, N.N., Bu, Y., Jin, Y., Qi, Y.leg, 26 September 2022. Four specimens in slides and numerous specimens in alcohol (SNHN): China, Guangxi Province, Nanning city, Wuxiangling Park (22°45'03.2"N 108°20'37.5"E), entomological aspirator, Godeiro, N.N., Bu, Y., Jin, Y., Qi, Y. leg, 27 September 2022. Three specimens in slides (two males and one female) (CC/UFRN): China, Guangxi Province, Nanning city, Wuxiangling Park (22°45'03.2"N 108°20'37.5"E), entomological aspirator, Godeiro, N.N., Bu, Y., Jin, Y., Qi, Y. leg, 27 September 2022. One specimen on slide, and two specimens in ethanol (SNHN): Taiwan, Penghu County, Magong City, Shuangtougua (23°33'58.4"N 119°35'22.1"E), collected by beating vegetations and entomological aspirator, 28 October 2023, HJ Cheng & DY Cheng leg. Two specimens (females) (CC/UFRN): Brazil, Rio Grande do Norte state, Natal municipality, Parque Estadual Dunas de Natal (Atlantic Forest biome) (5°49'12”S, 35°11'16”W), entomological aspirator, Simões, L.C.S. leg, November 2017. One specimen (female) (CC/UFRN): Brazil, Rio Grande do Norte state, Jardim do Seridó municipality, sítio Zangarelhas (Caatinga biome) (6°36'21,43"S 36°44'43,32"W), entomological aspirator, França, J.S. col & Siqueira, O.J.R. leg, 09 April 2018. One specimen (female) (CC/UFRN): Brazil, Rio Grande do Norte state, Nísia Floresta municipality, Lagoa Redonda Farm (Atlantic Forest biome) (06°02’45.02”S 35°11’42.63”W), 49m, pitfall trap, Paz, R.V & Carvalho, M.N.A leg., 15–16 April 2017.

Diagnosis based on the studied specimens. Lateral Th. II or Th. II–Abd. I with a dark purple longitudinal stripe, one distal spot on femora II–III, lateral Abd. III and latero-posterior Abd. IV–V dark purple spotted. Dorsal head **An** series with 7–8 mac, **A1**, **S2**, **S4** and **S6** as mic, with posterior head mac other than **Pa5**. Labial **r** chaeta smooth and reduced. Mesothoracic hood not developed, Th. II **a** (excluding the anterior collar), **m** and **p** series with 4, 7–8, 10–11 mac, **p5** mac present. Th. III–Abd. III central mac as 6–7, 2, 2–3, 1, respectively. Legs without any clear sexual dimorphism, tibiotarsus III without remarkably long chaetae, trochanteral organ with 24–34 spines, ungues lateral teeth regular, not enlarged, unguiculi acuminate, **p.e.** lamella slightly serrated. Ventral tube anteriorly with six ciliate chaetae and 4–5 proximal spines, posteriorly with 1–2 smooth regular chaetae and three spines, lateral flap with 11–13 chaetae, 4–13 smooth and 0–9 ciliate. Manubrium ventral chaetae formula from the basis to the subapical region as 1, 2, 2, 2/4, ventro-external subapical chaetae posterior to the internal ones, manubrial plate and dens lacking blunt mac.

Redescription based on the studied specimens. Studied specimens with 1.32–2.05 mm (n=6). Habitus typical of *Seira*. Specimens ground color pale or yellowish, antennae and tibiotarsi weakly purplish pigmented, lateral Th. II or Th. II–Abd. I with a dark purple longitudinal stripe, frontal head with a typical dark spot, one distal spot on femora II–III, lateral Abd. III and latero-posterior Abd. IV–V dark purple spotted. Scales present on Ant. I to proximal half of Ant. III, dorsal and ventral head, dorsal trunk, legs, anterior ventral tube, dorsal and ventral manubrium and ventral dens.

Head. Ratio Ant. I–Ant. IV as 1:1.3–2.5:1.5–2.8:2.3–4.4 (n=4). Ant. IV apical bulb distally bilobed (on lateral view apparently unilobed) (Fig. 7A), Ant. III apical organ with two sensory rods, three short guard sens, and at least six surrounding sens (Fig. 7B). Labral papillae present, internal ones conical and developed, lateral ones reduced (Fig. 7C). Labral **p**/**m**/**a** rows with 5/5/4 smooth chaetae respectively, **p** row chaetae slightly longer than the others, **a** row chaetae as thick as the **m** chaetae, pre-labral chaetae ciliated (Fig. 7D). Clypeal chaetotaxy as in Fig. 7E, all chaetae ciliate, **pf** chaetae as long as the **f** ones, **l1**–**2** long, strongly acuminate and slightly ciliate. Mandibles and maxillae without any clear modifications, labial papillae formula of the guards as **H**+2, **A**+0, **B**+5, **C**+0, **D**+4, **E**+4 plus a finger-shaped **l.p.** surpassing the apex of the papilla (Fig. 7F). Maxillary outer lobe **b.c.** subequal to **a.a.**, sublobal plate with four chaeta-like appendices (similar to Fig. 4G). Dorsal head with 7–8 **An**, 4 **A**, 2–3 **M**, 3 **S**, 5 **Pa**, 2 **Pm**, 3 **Pp** and 4 **Pe** mac; 8+8 eyes with 5 interocular chaetae, chaetal homologies and further details depicted in Fig. 8A. Labial proximal region with five smooth chaetae, labial basal chaetae formula as **M1**–**2rEL1**–**2**/**a1**–**5**; post labial region with ciliate chaetae, short smooth chaetae and scales, anterior region with chaetal formula **G1**–**4**/**H2**–**4**/**J1**–**4**, **H2** present or absent, **x** series with four scales, laterally with three short smooth chaetae, basal chaeta very long and slightly ciliate, further details depicted in Fig. 8B.

Trunk chaetotaxy. Mesothoracic hood not developed. Formula of the internal mac from Th. II (excluding the collar) to Abd. IV as 21–23, 6–7|2,2–3,1,8; of the external mac as: 1,1–2|0,1,3,11–13; and of the bothriotricha as 0,0|0,2,3,3. Abd. IV with 6–7 posterior mes next to about 6–7 psp; Abd. V with 11 mac; formulae of the tergal sens from Th. II to Abd. V as 1,1|0,2,2,+,3; and of **ms** as 0,0|1,0,1,0,0. All chaetal homologies and further details depicted in Figs 8C–D. Ratio Abd. III–IV in the midline as 1:2.9–4.0 (n=4).

Trunk appendages. Leg I without any clear sexual dimorphism. Coxae I–III with 3–4, 9–13, 9–12 ciliate chaetae and 2, 2–3, 2 psp, respectively, structures positioning as in Figs 9A–C. Trochanteral organ with 24–34 smooth spine-like chaetae disposed as in Fig. 9D. Tibiotarsi without remarkably long chaetae as seen in Neotropical *Lepidocyrtinus* taxa, tenent-hairs capitate and slightly ciliate; ungues with seven teeth, one dorsal, two paired lateral and four internal, lateral teeth not enlarged, **a.t.** reduced, **m.t.** smaller than **b.t.**; unguiculi lanceolate, **a.e.**, **a.i.**, **p.i.** lamellae smooth, **p.e.** slightly serrate, empodial complex III ratio of unguiculus, ungues, smooth chaeta and tenent-hair of one revised specimen as 1:1.7:1.1:2 (Fig. 9E). Ventral tube anteriorly scaled, with two distal mac, four long ciliate proximal chaetae and 4–5 smooth spines; posteriorly with three reduced smooth spines and 1–2 distal smooth chaetae; lateral flap with 11–13 chaetae, 4–13 smooth and 0–9 ciliate (Fig. 9F). Tenaculum rami with four teeth, corpus with a single ciliate chaeta. Manubrium ventral chaetae formula from the basis to the apex as 1, 2, 2, 2/4 (subapical) and 10–14 (apical) chaetae, subapical external pair of chaetae posterior to the internal ones (Fig. 9G). Manubrial plate with 4–5 regular ciliate chaetae, without blunt mac, plus three psp (Fig. 9H); dens without blunt mac; mucro typically falcate (similar to Fig. 6I). Ratio manubrium: mucrodens as 1.0:1.2–1.7 (n=4).

Variations. The main polymorphic traits of *S. brasiliana* shown in Figs 8, 9 were better observed in the Brazilian analyzed specimens like: head **M2** mac also as mic, Th. II **m4ip** and Th. III **a5** mic also as mac, Abd. II **m3** and Abd. IV **Fe3**–**4** mac absent, and the variations in trochanteral organ number of spines and ventral tube lateral flaps chaetotaxy, including the presence (or absence) of ciliate chaetae. Even so, the Chinese sampled specimens also have at least a variation in head **M2** mac (also as mic), number of chaetae on coxae I–III, and ventral tube anterior chaetotaxy. This observation suggests that *S. brasiliana* has some polymorphisms which likely induced the description of the species more than once.

For the remarks on the species, see the discussion topic.

***Seira ritae*** **Bellini & Zeppelini**[10]

*Seira ritae* Bellini & Zeppelini [10]: 403–405, figs 1–11, Brazil, Paraíba, João Pessoa, Praia do Bessa.

*Seira coroatensis* Godeiro & Bellini [45] **syn. nov.**

Notes. The type material of *S. ritae* was deposited at the National Museum of Rio de Janeiro (MNRJ) and was destroyed after a devastating fire in September 2018. The overall morphology and dorsal chaetae homology of the species are described in detail by Godeiro & Bellini [45]. A few differences listed between *S. coroatensis* **syn. nov.** and *S. ritae* in the remarks session of Godeiro & Bellini (45, pg 214) may be considered polymorphic traits of *S. ritae*. The color pattern and habitus presented in Bellini & Zeppelini (10, pg 404) for *S. ritae* possibly depicted a subadult specimen and are likely better represented in Godeiro & Bellini [45]. Even so, a variation in the color pattern of the species cannot be ruled out.

***Seira atrolutea*** ***(Arlé)*** [1]

*Pseudosira atrolutea* Arlé [1]: 297, figs 16–20, Brazil, São Paulo and Mato Grosso do Sul states.

*Seira paulae* Cipola & Bellini, 2014 in [28] **syn. nov.**

**Notes.** We could not track the type material of *S. atrolutea* at this time. However, the overall morphology and dorsal chaetae homology of the species are described in detail in Cipola et al. [28]. The differences in color pattern and unguiculus postero-external lamella (serrated *vs.* smooth) between *S. paulae* **syn. nov.** and *S. atrolutea* in the remarks session of Cipola et al. (28, pg 162) may be considered polymorphic traits of *S. atrolutea*. Other features, such as the real shape of Ant. IV apical bulb and absence of the tibiotarsus III distal smooth chaeta were likely mistakenly observed by Arlé [1].

## Discussion

### A Palaearctic origin for the Seirinae? An overview of recent phylogenetic findings

Recent studies concerning the Entomobryoidea have attempted to test the validity of its suprageneric taxa. The groundbreaking works of Zhang & Deharveng [108] and Zhang et al. [110] supported Seirinae as a valid and independent subfamily of Entomobryidae, detached from the *Lepidosira*-group of species. Nevertheless, a question remained unanswered in these studies: which of the other subfamilies was its sister group? The morphology of Seirinae taxa seems intermediate compared to that of Entomobryinae and Lepidocyrtinae [67, 90, 94, 99], and the sampling and analyses used until that time could not clearly determine which of these two subfamilies was the most closely related to Seirinae [108–111]. More recently, phylogenies based on mitogenomes of large datasets of Entomobryoidea supported a closer relationship between Seirinae and Lepidocyrtinae [15, 49], a result also achieved employing more robust markers, such as ultraconserved elements (UCEs) and universal single-copy orthologs (USCOs) [50]. These results are moreover endorsed by morphology at some extent [90, 94, 108].

Although a few representatives of Seirinae were included in recent phylogenies of the Entomobryoidea (as in [109–111]), the first study based on a more comprehensive dataset was presented by Godeiro et al. [46], aiming to study the Neotropical lineages of the group. In this study, 22 terminal taxa of Seirinae were sampled, and the main findings supported its current three genera: *Seira*, *Lepidocyrtinus* and *Tyrannoseira*. The inclusion of Asian and European species in newer phylogenies, however, put in check the monophyly of *Seira* [51], similarly to our achieved results (Fig. 2). The current knowledge on the subfamily, which is also represented by our phylogeny, points out to: European taxa may characterize the more basal lineages of Seirinae, but they were retrieved as polyphyletic; the sampled Asiatic taxa share a single ancestor (except for exotic species of course, see the next topic); and the Neotropical Seirinae, which gathers not only *Seira* but also *Tyrannoseira* and *Lepidocyrtinus*, form another monophyletic group within *Seira* s. lat. [52].

To date, we could not find any clear morphological features to circumscribe and clearly separate all the different lineages of *Seira* observed in our analyses. Jacquemart [57] proposed groups of species based on the disposition and number of dorsal macrochaetae on the trunk of *Seira*, with a simpler macrochaetotaxy observed in the *domestica-*group, and a more complex macrochaetotaxy (with a larger set of chaetae) in the *dollfusi*-group, especially regarding the thoracic segments. Although the notes of Jacquemart may first look simplistically to the current knowledge on *Seira* morphology, they may be useful to some extent for separating at least the European lineages, as *S. ferrarii* (from the *domestica-*group) was found in a distinct branch from *S. pallidipes* + *S. dollfusi* (both from the *dollfusi*-group). On the other hand, the separation of Asian species from Neotropical *Seira* is so far not evident from a morphological perspective, and we could not assign clear synapomorphies for each branch at this time. We believe that to better solve the phylogeny of the Seirinae and to ultimately identify potential traits able to split *Seira* into additional genera, some efforts still need to be made, especially: adding *S. domestica* to the analysis, since it is the type species of the genus and should delimit which taxa may actually fit in *Seira*; additionally, the species has sexual dimorphic legs somewhat similar to those of *Tyrannoseira*, although their dorsal chaetotaxy is remarkably different [9, 32]; and the addition of *Lepidocyrtinus* and *Seira* samples from Africa, which would help to test the validity of both genera, to determine the relationships among Neotropical, Afrotropical and Oriental taxa, and to better delimit a possible center of origin for the subfamily.

The Seirinae likely arose between the Middle and Upper Cretaceous or even before [50]. If recent findings on its evolution, including our tree, are accurate, the group emerged in the Palaearctic Region from an ancestor with moderate dorsal plurimacrochaetosis, such as *S. ferrarii*. This is an intriguing hypothesis since most species of the genus have a Holotropical distribution, with many species well adapted to hot climates [6, 8, 23, 57]. After this, distinct branches of Seirinae took different paths and gained further dorsal macrochaetae (as in the *dollfusi*-group), or lost many of them (as in some Neotropical lineages such as *S. brasiliana* and *Tyrannoseira*), and independently gained furcal blunt macrochaetae (like in the Asiatic *S. phrathongensis* **sp. nov.** and most Neotropical *Lepidocyrtinus*). Similarly, the modified first pair of legs of Neotropical *Tyrannoseira* males and some *Seira* species from the Old World, such as *S. domestica*, *S. mantis* Zeppelini & Bellini [104] and *S. uwei* Barra [5], likely arouse independently two times within the Seirinae as well [9, 29, 32].

### The recent colonization of Neotropical *Seira* in Asia

Although some Asian populations of Seirinae were found mixed within the Neotropical sampled group in our analysis (Fig. 2), our data indicate that these species are exotic taxa currently found in China and Thailand. These Asian populations were clustered within higher nodes of the Neotropical Seirinae, and they were identified as representatives of the Neotropical *S. dowlingi* and *S. brasiliana* (Fig. 2, Additional Files 2 and 3). Similar results were previously reported by Godeiro & Zhang [48] for Chinese and Brazilian populations of *S. dowlingi*, which share indistinguishable morphologies and remarkably similar mitogenomes. The high similarity rates of mitochondrial markers between distant populations, combined with their strong morphological resemblance, not only support that they belong to the same species but also that their introduction into new habitats was recent, likely due to human intervention [26]. Our tree also suggested that the Chinese populations of *S. brasiliana* sampled from the cities of Nanning and Xiamen (1,000 km apart) may have different origins, and the species may have been introduced more than once in China. However, such observation should be approached with caution, as the relationships between the internal branches of *S. brasiliana* populations had lower ML and BI support levels (Fig. 2). To verify this hypothesis, larger sample sizes and the inclusion of more informative markers at the population level would be required.

The recent accidental introduction of exotic springtails into new habitats through human activities appears to have been occurring extensively worldwide, and it has been documented at least to sub-Antarctic, European and African islands, Australia, New Zealand and North America [2, 26, 54, 82, 83]. Such invasions may have become more relevant since the Great Navigations era (15th century), with the exchange of living exotic plants (together with soil samples), fruits, seeds, cattle and other animals, timber and other commodities between different countries and continents. The more recent process of globalization has intensified the accidental introduction of alien species [75], and even with some countries’ protection policies against invasive biological components, there is evidence that springtail species are still being largely introduced into new ecosystems [54]. Our data concerning *S. dowlingi* and *S. brasiliana*, together with the data provided by Godeiro & Zhang [48], support the hypothesis that Neotropical species have recently invaded Asia, likely through human intervention as well. In this scenario, further studies should aim to investigate the extent of the current distribution of these taxa in Asia and other continents through a more detailed phylogeographic study, and the potential risks of such introductions to the native fauna.

### On *Seiradowlingi* identity

Before running our analyses, we were aware that Lima et al. [66] pointed to *S. musarum* Ridley [85] as a synonym of *S. dowlingi*. The authors made a good point based on biogeography of islands, considering that *S. musarum* is the sole species of *Seira* found widely across the Fernando de Noronha archipelago, Brazil, and its morphology is indistinguishable from that of *S. dowlingi*. The authors also declared that the type series of *S. musarum* could not be accessed at that time and was possibly lost [66]. However, Ridley’s [85] description of *S. musarum* is very brief and generic, and cannot clearly assign it as a *Seira* species. Therefore, although Lima et al. [66] may still be correct about the identity of *S. musarum*, in this uncertain scenario we chose to keep the name of *S. dowlingi* to our samples in this study, since for now it has a better resolved identity and is a well-studied species [90].

### Taxonomical remarks of *Seiraphrathongensis* sp. nov.

The morphology of the new species appears to be intermediate to that of the *Lepidocyrtinus* and *Seira* taxa [14, 33, 46, 99]. It has an extra group of anterior mac on Th. II (**a5i3** group), **p5** as a mic in the same tergite, and blunt mac on the manubrial plate and proximal dens, features that resemble many *Lepidocyrtinus* taxa [14, 33, 46, 99]. On the other hand, the species has all the main posterior mac on the dorsal head, a regular mesothoracic hood (mesonotum), underdeveloped and not projecting over the head, and ungues lateral teeth regular sized, not enlarged, features more fitting to *Seira s. str.* taxa [33, 46, 90]. This intermediate morphology between both genera, and especially the underdeveloped mesothoracic hood and furca bearing only a few blunt mac, resembles the concept of *Austroseira* Yoshii & Suhardjono [103], a group which was already synonymized with *Lepidocyrtinus* by Cipola et al. [33]. Even so, the new species was herein kept as a *Seira* taxon based on our phylogenetic tree, which places it outside the sampled *Lepidocyrtinus* taxa (Fig. 2).

The closest species to *Seira phrathongensis* **sp. nov.** is *L. schaefferi* (Schött) [88] *sensu* Gapud [42]. They share a somewhat similar color pattern and comparable overall chaetotaxy (see Table 3), including the presence of six mac on Abd. I, blunt mac on the manubrial plate and on the dorsoproximal dens. However, *Seira phrathongensis* **sp. nov.** differs from *L. schaefferi* especially in the following: coxae II and III without pigments (present in *L. schaefferi*; presence of **Pa2** and **Pa4** (**S6i** eventually) mac on the posterior dorsal head (all absent in *L. schaefferi*); labral external papillae clearly smaller than the internal ones (subequal in *L. schaefferi*); ventral tube lateral flap with up to 13 smooth chaetae, or with at most two ciliate chaetae (with 15 chaetae, seven of them ciliate in *L. schaefferi*), and manubrial plate with two blunt mac (*vs.* at least three in *L. schaefferi*). The depiction of *L. schaefferi* by Yoshii & Suhardjono [103], which shows more details on the species, also adds further differences between the species, such as Th. II **a5i3** complex with four mac and **m4p** and **p5** mac present (*vs.* three mac on **a5i3** complex and **m4p** and **p5** absent in *L. schaefferi*), and manubrial ventro-external subapical chaetae posterior or aligned to the internal ones in *Seira phrathongensis* **sp. nov.** (*vs.* anterior in *L. schaefferi*).

The similarities between the new species and *L. schaefferi* may imply that the latter would also better fit in *Seira*. However, since we did not access *L. schaefferi* samples for our BI/ML analyses and the phylogeny of the Seirinae was partially unsolved, we rather keep it provisionally in *Lepidocyrtinus*. It is also worth noting that the depiction of Yoshii & Suhardjno [103] of *L. schaefferi* based on Indonesian specimens shows some important differences in the macrochaetotaxy of Th. II–Abd. I and manubrium compared to the Philippine specimens of Gapud [42], including the number of mac on the Abd. I (see Table 3). This observation combined with the wide distribution of *L. schaefferi* may suggest that it is actually a complex of species.

Considering the Thai fauna of *Seira*, *S. phrathongensis* **sp. nov.** shares with *S. thailandica* the presence of six mac on Abd. I. Although the latter species description lack many important data [101], it can be readily separated from the new species especially by: Th. II posteriorly with 14 mac (*vs.* 15 in *S. phrathongensis* **sp. nov.**; trochanteral organ with about 12 spines (*vs.* 18–19); ventral tube anterior face with only two ciliate chaetae (*vs.* eight); posterior face with six spines, with two of them unpaired (*vs.* two, none of them unpaired); and more importantly, absence of blunt chaetae on the furca (*vs.* presence). The habitat and distribution of both species also differ, where the new species was found on an orchard on the island in the southern region, near sea level (10 meters asl.), while *S. thailandica* was collected from the highest mountain of the country, in Doi Inthanon (2,565 meters asl.), in a forested area in northern Thailand. Further comparisons between the Asiatic species of Seirinae with six mac on the Abd. I are presented in Table 3.

### *Seirabrasiliana* morphology and distribution

Our redescription of *S. brasiliana* mostly complies with the depictions of Arlé [1], Mari-Mutt [70], Lee & Park [65], Christiansen & Bellinger [23], Soto-Adames [90], and Bellini et al. [12]. Some polymorphic chaetae on the dorsal body presented by other authors were not observed in our analyzed material, like on head, internal Th. II–III, Abd. II and Abd. IV in Mari-Mutt [70]; internal Th. II–III and Abd. II of Christiansen & Bellinger [23]; and internal Th. II of Bellini et al. [12], supporting the dorsal macrochaetotaxy of the species is quite labile, similarly to its color pattern [1]. Our redescription also corrects mistakes seen in Bellini et al. [12], such as the shape of the labral papillae (only the labral ornamentations were drawn in the description of *S. potiguara*), the position of the unguiculi serrations (also represented to **a.i.** lamella) and unguiculi shape (acuminate instead of truncate). The absence of unguiculi serrations in **p.e.** lamella in the drawings of Arlé [1] and Mari-Mutt [70], and as observed by Lee & Park [65], may indicate another polymorphic trait of the species, although due to the size, such serrations can be easily overlooked.

*Seira brasiliana* is widely distributed in the Neotropical Region, being recorded from the Caribbean (Puerto Rico, US Virgin Islands and the Lesser Antilles), Brazil, Bolivia and Florida, USA [23, 71, 72], mostly in regions with a main equatorial climate [61]. In Brazil, the largest country of South America, the species was recorded in the southern, central-western, southeastern and northeastern regions, from three distinct phytogeographic domains (Cerrado, Caatinga and Atlantic Forest), supporting it is also widely distributed throughout the country [106]. In China, its representatives were collected in urban forest parks with high vegetation cover. A somewhat similar widespread distribution is also observed for *S. dowlingi* in the Americas [32]. Interestingly, these are the two Neotropical species herein reported to Asia. Both taxa support a wide range of different habitats, including some disturbed by anthropogenic activities. For instance, specimens of *S. brasiliana* were collected from soybean and sugarcane crops in Puerto Rico by Mari-Mutt [70], and we collected specimens from a recreational plaza and from lettuce and cilantro crops in Rio Grande do Norte state, Brazil (personal observation). Similarly, specimens of *S. dowlingi* were collected in a sugarcane mill in Puerto Rico [90], as well as in urban areas, including rooms in apartments, in Amazonas state, Brazil [32]. Such observations suggest that these species are resilient to habitat modifications, at least to some extent, which is an ideal condition for allowing them to invade new habitats.

## Conclusions

Seirinae represents a major component of the epedaphic fauna of springtails in tropical and subtropical environments. In this study we were able to contribute to the understanding of its internal relationships, adding further taxa to the tree of the group, and reviewing recent findings on its phylogenetics. We could also identify and suggest four synonyms for previously described species, pointing out for the first record of *Seira brasiliana* outside the Americas. This species morphology was herein redescribed in detail, as well as the new *Seira phrathongensis* **sp. nov.** from Thailand. Through these efforts, we tried to provide further ground on the understanding of the evolutionary history, systematics, distribution and taxonomy of the group, contributing to future studies on Seirinae and Entomobryidae. We believe that the next major step regarding the subfamily systematics is splitting it into additional genera.

## Supplementary Information

**Additional file 1.** ModelFinder for ML analyses. Substitution models selected by ModelFinder used for ML analyses with 13 partitions. Complementary data used before performing ML analyses, as explained in the Methods topic.

**Additional file 2.** Delimitation results of bPTP analysis. Delimitation results of bPTP analysis. Achieved OTUs in this study according to the bPTP analysis, as detailed in the Results topic.

**Additional file 3.** MEGA dist. Estimates of Evolutionary Divergence between Sequences. The number of amino acid differences per sequence from between sequences are shown. This analysis involved 28 amino acid sequences. All positions with less than 5% site coverage were eliminated, i.e., fewer than 95% alignment gaps, missing data, and ambiguous bases were allowed at any position. There were a total of 3465 positions in the final dataset. Evolutionary analyses were conducted in MEGA X. Divergence between sequences of the samples used to delimit the valid species, to double-check the bPTP delimitation results, as detailed in the Methods and Results topics.

## Data Availability

The mitochondrial genomes of *Seira phrathongensis* **sp. nov.**, *S. brasiliana* (Nanning) and *S. brasiliana* (Xiamen) are available in the GenBank National Center for Biotechnology Information (NCBI) nucleotide database under accession numbers PP191133.1, OR804098.1, and OR804097.1, respectively. The raw Illumina data were deposited in the NCBI SRA database under accession numbers SRR27031806, SRR27031805, and SRR24733363 for *Seira phrathongensis* **sp. nov.**, *S. brasiliana* (Nanning) and *S. brasiliana* (Xiamen), respectively. Data on other samples used in our analyses are listed in Table 1. The studied specimens for morphological depictions and comparisons are deposited at CC/UFRN, INPA, NHM-PSU and SNHM.

## Abbreviations

Abd.: Abdominal segment(s)
als.: Above sea level
Ant.: Antennal segment(s)
mac: Macrochaeta(e)
mes: Mesochaeta(e)
mic: Microchaeta(e)
ms: Specialized microchaeta(e)
psp: Pseudopore(s)
sens: Specialized ordinary chaeta(e)
Th.: Thoracic segment(s), for head and mouthparts
b.c.: Basal chaeta
a.a.: Apical appendage
l.p.: Lateral process of labial papilla E
l.p.c.: Labial proximal chaeta(e), for unguis
a.t.: Apical tooth
b.t.: Basal teeth
m.t.: Median tooth for unguiculus
a.e.: Antero-external lamella
a.i.: Antero-internal lamella
p.i.: Postero-internal lamella
p.e.: Postero-external lamella biological material collections
CC/UFRN: The Collembola Collection of the Federal University of Rio Grande do Norte, Rio Grande do Norte, Brazil
INPA: The Invertebrate Collection of the National Institute of Amazonian Research, Manaus, Brazil
NHM-PSU: The Princess Maha Chakri Sirindhorn Natural History Museum, Songkhla, Thailand
SNHM: The Natural History Museum, Shanghai, China
BI: Bayesian inference
ML: Maximum likelihood
