# Supplementary material for: Exploring mitogenomic data to enhance the understanding of Seirinae (Collembola: Entomobryidae) evolution, distribution and taxonomy
Source: Front Zool. 2024 Dec 4;21:31. doi: 10.1186/s12983-024-00549-9 (PMC11616167; doi:10.1186/s12983-024-00549-9)
Supplement: Supplementary file 1 — Additional file 1. ModelFinder for ML analyses. Substitution models selected by ModelFinder used for ML analyses with 13 partitions. Complementary data used before performing ML analyses, as explained in the Methods topic. [file 12983_2024_549_MOESM1_ESM.docx]

|  | **Gene** | **AA sites** | **Informative** | **Invariants** | **Model** |
| --- | --- | --- | --- | --- | --- |
| 1 | ATP6 | 147 | 61 | 75 | mtMet |
| 2 | ATP8 | 54 | 44 | 9 | mtInv |
| 3 | COX1 | 510 | 81 | 392 | mtZOA |
| 4 | COX2 | 225 | 87 | 127 | mtMet |
| 5 | COX3 | 261 | 91 | 147 | mtART |
| 6 | CYTB | 377 | 127 | 182 | mtMet |
| 7 | ND1 | 292 | 123 | 135 | mtInv |
| 8 | ND2 | 318 | 239 | 55 | mtInv+F |
| 9 | ND3 | 112 | 56 | 42 | mtMet |
| 10 | ND4 | 396 | 218 | 125 | mtInv |
| 11 | ND4L | 84 | 54 | 25 | mtInv |
| 12 | ND5 | 548 | 314 | 194 | mtInv |
| 13 | ND6 | 141 | 104 | 26 | mtMet+F |

**Additional File 1.** Substitution models selected by ModelFinder used for ML analyses with 13 partitions.
