# Supplementary material for: Exploring mitogenomic data to enhance the understanding of Seirinae (Collembola: Entomobryidae) evolution, distribution and taxonomy
Source: Front Zool. 2024 Dec 4;21:31. doi: 10.1186/s12983-024-00549-9 (PMC11616167; doi:10.1186/s12983-024-00549-9)
Supplement: Supplementary file 2 — Additional file 2. Delimitation results of bPTP analysis. Delimitation results of bPTP analysis. Achieved OTUs in this study according to the bPTP analysis, as detailed in the Results topic. [file 12983_2024_549_MOESM2_ESM.docx]

| **OTUs** | **PP** | **Taxa name** |
| --- | --- | --- |
| 1 | 1.00 | *Seira ferrarii* |
| 2 | 1.00 | *Seira sanloemensis* |
| 3 | 1.00 | *Lepidocyrtinus paraibensis* |
| 4 | 1.00 | *Lepidocyrtinus dapeste* |
| 5 | 1.00 | *Seira boneti* |
| 6 | 1.00 | *Seira phrathongensis* **sp. nov.** |
| 7 | 1.00 | *Tyrannoseira bicolorcornuta* |
| 8 | 1.00 | *Tyrannoseira raptora* |
| 9 | 0.72 | *Seira dowlingi* BRA*, Seira dowlingi* THA*,Seira dowlingi* MEX*, Seira dowlingi* CH |
| 10 | 1.00 | *Seira tinguira* |
| 11 | 1.00 | *Lepidocyrtinus harena* |
| 12 | 1.00 | *Lepidocyrtinus diamantinae* |
| 13 | 0.94 | *Seira oligoseta* NAN-CH*,Seira brasiliana* PR-BR*, Seira potiguara* RN-BR*, Seira oligoseta* XIA-CH |
| 14 | 1.00 | *Seira dollfusi* |
| 15 | 1.00 | *Seira pallidipes* |
| 16 | 0.99 | *Seira atrolutea, Seira paulae* |
| 17 | 0.93 | *Seira coroatensis, Seira ritae* |
| 18 | 0.96 | *Seira mendoncae* |

**Additional File 2.** Delimitation results of bPTP analysis (OTUs – operational taxonomic units; PP – posterior probability).
