# Supplementary material for: Exploring mitogenomic data to enhance the understanding of Seirinae (Collembola: Entomobryidae) evolution, distribution and taxonomy
Source: Front Zool. 2024 Dec 4;21:31. doi: 10.1186/s12983-024-00549-9 (PMC11616167; doi:10.1186/s12983-024-00549-9)
Supplement: Supplementary file 3 — Additional file 3. MEGA dist. Estimates of Evolutionary Divergence between Sequences. The number of amino acid differences per sequence from between sequences are shown. This analysis involved 28 amino acid sequences. All positions with less than 5% site coverage were eliminated, i.e., fewer than 95% alignment gaps, missing data, and ambiguous bases were allowed at any position. There were a total of 3465 positions in the final dataset. Evolutionary analyses were conducted in MEGA X. Divergence between sequences of the samples used to delimit the valid species, to double-check the bPTP delimitation results, as detailed in the Methods and Results topics. [file 12983_2024_549_MOESM3_ESM.docx]

|  |  | 1 | 2 | 3 | 4 | 5 | 6 | 7 | 8 | 9 | 10 | 11 | 12 | 13 | 14 | 15 | 16 | 17 | 18 | 19 | 20 | 21 | 22 | 23 | 24 | 25 | 26 | 27 | 28 |
| --- | --- | --- | --- | --- | --- | --- | --- | --- | --- | --- | --- | --- | --- | --- | --- | --- | --- | --- | --- | --- | --- | --- | --- | --- | --- | --- | --- | --- | --- |
| 1 | *Lepidocyrtus sotoi* |  |  |  |  |  |  |  |  |  |  |  |  |  |  |  |  |  |  |  |  |  |  |  |  |  |  |  |  |
| 2 | *Seira oligoseta* Nan | 1004 |  |  |  |  |  |  |  |  |  |  |  |  |  |  |  |  |  |  |  |  |  |  |  |  |  |  |  |
| 3 | *Seira coroatensis* | 969 | 389 |  |  |  |  |  |  |  |  |  |  |  |  |  |  |  |  |  |  |  |  |  |  |  |  |  |  |
| 4 | *Seira dollfusi* | 613 | 549 | 519 |  |  |  |  |  |  |  |  |  |  |  |  |  |  |  |  |  |  |  |  |  |  |  |  |  |
| 5 | *Seira oligoseta* | 999 | 69 | 388 | 544 |  |  |  |  |  |  |  |  |  |  |  |  |  |  |  |  |  |  |  |  |  |  |  |  |
| 6 | *Seira sanloemensis* | 797 | 676 | 667 | 448 | 675 |  |  |  |  |  |  |  |  |  |  |  |  |  |  |  |  |  |  |  |  |  |  |  |
| 7 | *Seira dowlingi* BRA | 892 | 586 | 575 | 451 | 586 | 575 |  |  |  |  |  |  |  |  |  |  |  |  |  |  |  |  |  |  |  |  |  |  |
| 8 | *Seira dowlingi* CH | 934 | 605 | 592 | 518 | 604 | 615 | 70 |  |  |  |  |  |  |  |  |  |  |  |  |  |  |  |  |  |  |  |  |  |
| 9 | *Tyrannoseira bicolorcornuta* | 1022 | 832 | 818 | 579 | 837 | 675 | 740 | 766 |  |  |  |  |  |  |  |  |  |  |  |  |  |  |  |  |  |  |  |  |
| 10 | *Seira dowlingi* MEX | 969 | 668 | 650 | 559 | 668 | 652 | 54 | 90 | 842 |  |  |  |  |  |  |  |  |  |  |  |  |  |  |  |  |  |  |  |
| 11 | *Seira dowlingi* THA | 1002 | 655 | 636 | 555 | 654 | 658 | 6 | 69 | 830 | 53 |  |  |  |  |  |  |  |  |  |  |  |  |  |  |  |  |  |  |
| 12 | *Lepidocyrtinus harena* | 1024 | 984 | 940 | 624 | 985 | 701 | 886 | 887 | 1038 | 977 | 978 |  |  |  |  |  |  |  |  |  |  |  |  |  |  |  |  |  |
| 13 | *Seira pallidipes* | 988 | 1022 | 996 | 197 | 1013 | 743 | 905 | 910 | 1061 | 999 | 1006 | 1095 |  |  |  |  |  |  |  |  |  |  |  |  |  |  |  |  |
| 14 | *Seira boneti* | 1009 | 990 | 949 | 573 | 988 | 638 | 877 | 889 | 1001 | 987 | 985 | 1025 | 1050 |  |  |  |  |  |  |  |  |  |  |  |  |  |  |  |
| 15 | *Seira atrolutea* | 988 | 389 | 328 | 547 | 394 | 681 | 560 | 597 | 830 | 653 | 639 | 981 | 1025 | 977 |  |  |  |  |  |  |  |  |  |  |  |  |  |  |
| 16 | *Lepidocyrtinus dapeste* | 1020 | 1035 | 985 | 605 | 1029 | 750 | 923 | 917 | 1057 | 1020 | 1019 | 744 | 1131 | 1033 | 1027 |  |  |  |  |  |  |  |  |  |  |  |  |  |
| 17 | *Lepidocyrtinus paraibensis* | 1031 | 975 | 945 | 597 | 973 | 680 | 857 | 867 | 990 | 952 | 957 | 962 | 1077 | 1000 | 963 | 1028 |  |  |  |  |  |  |  |  |  |  |  |  |
| 18 | *Lepidocyrtinus diamantinae* | 1020 | 980 | 940 | 585 | 982 | 695 | 877 | 883 | 1017 | 978 | 978 | 437 | 1062 | 1008 | 967 | 734 | 952 |  |  |  |  |  |  |  |  |  |  |  |
| 19 | *Seira mendoncae* | 989 | 386 | 218 | 542 | 390 | 677 | 561 | 594 | 825 | 645 | 633 | 977 | 1017 | 963 | 337 | 1011 | 945 | 974 |  |  |  |  |  |  |  |  |  |  |
| 20 | *Seira phrathongensis* sp n | 1026 | 988 | 945 | 577 | 986 | 636 | 863 | 869 | 1021 | 966 | 968 | 1024 | 1046 | 731 | 985 | 1064 | 1033 | 1037 | 966 |  |  |  |  |  |  |  |  |  |
| 21 | *Lepidocyrtus nigrosetosus* | 548 | 969 | 926 | 549 | 969 | 683 | 910 | 850 | 979 | 958 | 959 | 1014 | 935 | 989 | 940 | 976 | 989 | 991 | 946 | 984 |  |  |  |  |  |  |  |  |
| 22 | *Seira ferrarii* | 953 | 977 | 945 | 557 | 978 | 700 | 899 | 915 | 973 | 960 | 971 | 998 | 991 | 974 | 984 | 1036 | 996 | 994 | 971 | 978 | 912 |  |  |  |  |  |  |  |
| 23 | *Seira tinguira* | 970 | 444 | 439 | 534 | 447 | 648 | 577 | 593 | 847 | 665 | 649 | 970 | 1016 | 967 | 432 | 999 | 955 | 961 | 434 | 967 | 922 | 959 |  |  |  |  |  |  |
| 24 | *Tyrannoseira raptora* | 1027 | 874 | 876 | 599 | 875 | 723 | 785 | 796 | 641 | 895 | 883 | 1071 | 1088 | 1034 | 873 | 1092 | 1012 | 1059 | 881 | 1069 | 1011 | 1026 | 874 |  |  |  |  |  |
| 25 | *Seira brasiliana* | 981 | 41 | 387 | 527 | 73 | 664 | 587 | 600 | 819 | 663 | 649 | 966 | 1002 | 969 | 384 | 1013 | 957 | 963 | 381 | 968 | 938 | 957 | 444 | 863 |  |  |  |  |
| 26 | *Seira potiguara* | 980 | 30 | 384 | 531 | 63 | 659 | 583 | 596 | 820 | 658 | 644 | 972 | 1002 | 966 | 383 | 1017 | 962 | 966 | 382 | 967 | 946 | 959 | 444 | 861 | 35 |  |  |  |
| 27 | *Seira paulae* | 987 | 390 | 326 | 550 | 396 | 682 | 558 | 596 | 830 | 652 | 637 | 984 | 1023 | 977 | 16 | 1028 | 963 | 967 | 336 | 983 | 940 | 985 | 430 | 871 | 385 | 384 |  |  |
| 28 | *Seira ritae* | 973 | 393 | 45 | 521 | 392 | 668 | 578 | 595 | 818 | 653 | 640 | 943 | 998 | 951 | 337 | 990 | 947 | 941 | 224 | 946 | 923 | 942 | 439 | 877 | 392 | 389 | 332 |  |

**Additional File 3.** Estimates of Evolutionary Divergence between Sequences. The number of amino acid differences per sequence from between sequences are shown. This analysis involved 28 amino acid sequences. All positions with less than 5% site coverage were eliminated, i.e., fewer than 95% alignment gaps, missing data, and ambiguous bases were allowed at any position (partial deletion option). There were a total of 3465 positions in the final dataset. Evolutionary analyses were conducted in MEGA X [1][2]

| 1. Kumar S., Stecher G., Li M., Knyaz C., and Tamura K. (2018). MEGA X: Molecular Evolutionary Genetics Analysis across computing platforms. Molecular Biology and Evolution 35:1547-1549. |
| --- |
| 2. Stecher G., Tamura K., and Kumar S. (2020). Molecular Evolutionary Genetics Analysis (MEGA) for macOS. Molecular Biology and Evolution (https://doi.org/10.1093/molbev/msz312). |
